# Ribosomal RNA processing undergoes surveillance by the mRNA guard protein Npl3

**DOI:** 10.1007/s00018-026-06246-6

**Published:** 2026-05-19

**Authors:** Anne-Sophie Lindemann, Fei Yu, Yawen Duan, Ivo Coban, Ulla-Maria Schneider, Jan-Philipp Lamping, Ali Khreiss, Katherine E. Bohnsack, Heike Krebber

**Affiliations:** 1 Abteilung Für Molekulare Genetik, Institut Für Mikrobiologie Und Genetik, Göttinger Zentrum Für Molekulare Biowissenschaften (GZMB), Georg-August Universität Göttingen, Göttingen, Germany; 2 Institut Für Molekularbiologie, Universitätsmedizin Göttingen, Göttingen, Germany

**Keywords:** RRNA maturation, RRNA processing, RNA quality control, Ribosome biogenesis, RNA degradation, RDNA, RNA polymerase I, TRAMP complex, RNA exosome, 23S rRNA, ETS1

## Abstract

Ribosome production is an essential, but highly energy-demanding and complex cellular process. High-fidelity ribosome assembly is critical to ensure integrity of the proteome, and during their biogenesis, the pre-ribosomal subunits undergo surveillance so that defective complexes are removed. Currently, knowledge on the mechanisms of pre-ribosome quality control lag behind understanding of other RNA surveillance pathways. Interestingly, a family of “guard proteins” has been shown to monitor mRNA maturation in *S. cerevisiae* and act as maturation switches by either licensing ongoing biogenesis or, in case assembly defects are detected, recruiting components of the RNA degradation machinery. Here, we reveal association of the mRNA guard protein Npl3 with RNA polymerase I, the ribosomal DNA (rDNA) locus and early pre-ribosomal particles. Consistent with this, our data show that Npl3 is required for recruitment of a subset of ribosome assembly factors to the nascent pre-rRNA transcript, and we demonstrate a role for Npl3 in the turn-over of aberrant precursor ribosomal RNA (pre-rRNA). Aberrant pre-rRNAs polyadenylated by Trf5 are bound by Npl3, which in turn interacts with Air1 to recruit the RNA exosome for degradation. Thus, Npl3 is a multifunctional RNA surveillance factor, recognizing different types of aberrant pre-RNAs and promoting recruitment of the RNA degradation machinery to clear these transcripts.

**Supplementary Information:**

The online version contains supplementary material available at 10.1007/s00018-026-06246-6.

## Introduction

Ribosomes are conserved and essential across the domains of life as they produce all cellular proteins. Although protein synthesis takes place in the cytoplasm, the assembly of cytosolic ribosomes starts in the nucleolus in all eukaryotes [1, 2]. RNA polymerase I (RNAPI)-mediated transcription of the ribosomal DNA (rDNA) produces a precursor ribosomal RNA (pre-rRNA) that is processed to yield three rRNAs (18S, 5.8S and 25S (yeast)/28S (human)) that are bound by approximately 80 ribosomal proteins to form the small and the large ribosomal subunits (SSU and LSU, respectively). The Baker’s yeast (*Saccharomyces cerevisiae*, hereafter termed yeast) is a useful model system and has been extensively used to elucidate the fundamental principles of ribosomal subunit assembly. In yeast, around 200 non-ribosomal assembly factors contribute to the processing, folding and maturation of the rRNAs as well as correct assembly of the ribosomal proteins [3]. Due to its complexity, this process requires tight regulation to prevent production of aberrant ribosomal subunits. Accurate subunit assembly is essential for proper ribosome function, which is, in turn, crucial for faithful protein synthesis, cell function and organismal health [4]. Therefore, surveillance mechanisms for pre-rRNA processing and ribosomal subunit maturation are of paramount importance, but the underlying mechanisms are far from being understood.

While relatively little is known about quality control of ribosomal subunit assembly, various mRNA surveillance pathways have been identified and some factors involved have been characterized in detail. For example, several guard proteins have emerged as key mediators of mRNA quality control. These RNA-binding proteins are co-transcriptionally loaded onto each pre-mRNA by RNA polymerase II (RNAPII), and they recruit the export receptor heterodimer Mex67-Mtr2 upon successful 5’ capping, splicing, 3’ cleavage and polyadenylation, thus promoting nuclear export of the matured mRNA. However, in case any maturation events do not occur correctly, the guard proteins become retention factors that recruit the degradation machinery instead of nuclear export factors to promote degradation of the faulty pre-mRNA [5]. More specifically, the guard protein Npl3 was shown to survey 5’ capping [6–8] and to influence 3’ cleavage [9–11], whereas the guard proteins Gbp2 and Hrb1 monitor pre-mRNA splicing [8, 12–14], Hrp1 senses proper 3’ cleavage [15] and Nab2 is responsible for controlling correct polyadenylation [8, 16–18]. Mechanistically, the guard protein Npl3 monitors pre-mRNA capping via binding of the cap binding complex (CBC) to the 5’ end of the pre-mRNA. Upon interaction of Npl3 with the CBC, Mex67 binds to Npl3 and the mRNA is licensed for nuclear export. On defective caps, the interaction of Npl3 with Rai1 is stabilized, leading to recruitment of the 5’−3’ exoribonuclease Rat1 [6]. Upon contact with Rai1, Rat1 is activated and promotes degradation of the faulty pre-mRNA. Interestingly, RNA binding by Npl3 is not restricted to mRNAs as this protein has also been shown to associate with other classes of RNAs, including rRNAs [9, 12]. It is conceivable, therefore, that Npl3 may monitor also pre-rRNA processing events.

Pre-rRNA is transcribed by RNAPI, which, in yeast, is composed of 14 subunits, including the large subunits Rrp190 and Rrp135 and the two non-essential components Rpr49 and Rrp14 [19]. The polycistronic pre-rRNA, 35S, is composed of the 18S, 5.8S and 25S rRNAs that are flanked by two external transcribed spacers (5’ ETS and 3’ ETS) and interspersed by two internal transcribed spacers (ITS1 and ITS2). These spacers are progressively removed through a hierarchical series of endoribonucleolytic cleavage events and exoribonucleolytic trimming steps. The 3’end of the 25S rRNA is produced through cleavage by the endoribonuclease Rnt1 within the 3’ ETS to terminate transcription [2, 20]. In 70—80% of fast-growing yeast cells, co-transcriptional cleavage within ITS1 occurs prior to cleavage at the 3’end of the 25S rRNA [21–23]. Pre-rRNA processing typically occurs stepwise, with initial cleavages at sites A_0_ and A_1_ in the 5’ ETS and at A_2_ within ITS1 [2]. This results in formation of the 20S and 27S-A_2_ pre-rRNAs, which after additional processing, are destined for the small, and large subunit (SSU and LSU), respectively. The 20S pre-rRNA is exported into the cytoplasm where final processing at the 3’ end generates the 18S rRNA component of the SSU. The 27S pre-rRNA can be processed via alternative pathways, both of which result in the 5.8S and 25S rRNAs of the LSU. The processing pathways of the 5.8S and 25S rRNAs include trimming steps from the 5’ ends that involves the exoribonuclease Rat1 and 5.8S maturation also involves processing from the 3’ end, which is mediated by the exosome [24].

The initial pre-rRNA processing steps are crucial for ribosomal subunit assembly and defects can occur when the recruitment of ribosomal proteins and/or assembly factors is impaired [25–27]. The SSU processome is the first pre-ribosomal particle formed, and it assembles co-transcriptionally on the nascent pre-rRNA transcript. Assembly of the SSU processome is a modular process in which a series of subcomplexes are recruited in a hierarchical order. The UTP-A (Utp4, 5, 8, 9, 10, 15, 17 and Pol5), UTP-B (Utp1, 6, 12, 13, 18, 21) and UTP-C (Utp22, Rrp7 and CKII) complexes are sequentially assembled, before the Bms1-Rcl1 complex associates [28]. These binding events are coordinated through the U3 snoRNP and the Mpp10-Imp3-Imp4 complex. With correct SSU processome assembly, pre-rRNA cleavage occurs at A_0_ by a so far unknown enzyme, and thereafter at A_1_ and A_2_ likely by Utp24, before the RNase MRP cleaves at A_3_ [29, 30]. Defects in SSU processome assembly delay cleavage at A_0_, A_1_ and/or A_2_, resulting in an RNase MRP-mediated A_3_ cleavage, which is independent and inevitable, and leads to formation of the faulty 23S pre-rRNA [31]. Thus, the aberrant 23S pre-rRNA is produced when prior pre-rRNA cleavages were not executed [25, 26]. Importantly, the 23S pre-rRNA is a dead-end product that cannot undergo further processing, and as such, is eliminated by the pre-rRNA surveillance system, involving the TRAMP and RNA exosome complexes [32]. How the TRAMP-complex recognizes the 23S rRNA is unknown, however it marks this aberrant transcript with an oligo(A) tail, which exceeds the usual 4—5 nucleotide length, for recognition by the nuclear Rrp6-containing exosome leading to its degradation [26, 33]. The TRAMP-complex consists of either Trf4 or Trf5, non-cannonical poly(A) polymerases, as well as Air1 or Air2, which are two RNA-binding proteins suggested to connect Trf4 or Trf5 to RNA substrates and the helicase Mtr4, which positions RNA for entrance into the exosome [34]. The TRAMP4 complex contains Trf4, Air2 and Mtr4, whereas the TRAMP5 complex is comprised of Trf5, Air1 and Mtr4. To some extent, the proteins of TRAMP4 and TRAMP5 seem to be functionally redundant and interchangeable; both are involved in mRNA and rRNA degradation. However, only TRAMP5 binds to the sequence between A2 and A3 in the ITS1 of the rRNA and polyadenylates the A_2_-A_3_ product and the 23S rRNA [35].

Interestingly, the depletion of *NPL3* leads to accumulation of the 23S pre-rRNA [36], suggesting that this guard protein might play a role in pre-rRNA surveillance, similar to its function in mRNA quality control. Here, we uncovered a novel function for Npl3 in ribosomal subunit assembly and pre-rRNA surveillance. We show that it is involved in ensuring the timely recruitment of SSU processome proteins to the pre-rRNA. Impaired pre-rRNA association of some SSU processome components in *npl3∆* cells leads to accumulation of the faulty 23S pre-rRNA, which is oligoadenylated by the TRAMP5 complex and subsequently degraded by the exosome. Additionally, we demonstrate that Npl3-mediated loading of Air1 provides the necessary pre-ribosome entry points for assembly of the TRAMP5 complex and efficient degradation of the 23S pre-rRNA by the exosome. Taken together, our data reveal that Npl3 is a multifunctional guard protein that connects mRNA surveillance with pre-rRNA quality control.

## Material and methods

### Yeast strains, plasmids and oligonucleotides

Most yeast strains used in this study are based on *S. cerevisiae BY4741* and few strains were based on *S288C* (herein referred to as “wild type” despite the presence of the auxotrophic markers *URA3*, *LEU2* and *HIS3*) listed in Supplementary Table S1, plasmids in Supplementary Table S2 and oligonucleotides in Supplementary Table S3—8. Strains were cultivated in standard media at 25 °C [37, 38]. All newly created double-mutant strains were generated by crossing the respective parental strains of the opposite mating type [38]. Diploid strains were then sporulated and subjected to tetrad dissection. All haploid spores were analyzed according to their genetic markers. Endogenous Myc- or GFP-tagging was conducted by transforming a wild type yeast strain with a PCR product containing the tag as well as a *URA3* marker, flanked by loxP sites and the homologues sequences for integration into the locus of interest. For the generation of plasmids, conventional molecular biology methods were used.

### Growth analyses

Yeast cells were grown to log phase (1—2 × 10^7^ cells/ml) and diluted to 1 × 10^7^ cells/ml. Growth was either determined in liquid culture upon addition of 200 ng/ml rapamycin by counting the cells or on plates in drop dilution assays. For this purpose, ten-fold serial dilutions to 1 × 10^3^ cells/ml were prepared and 7 μl of each dilution was spotted onto either full medium (YPD) agar plates, selective plates, FOA plates, or plates containing 200 ng/ml rapamycin. The plates were incubated for 3 days at the indicated temperatures. Pictures were taken after the indicated days with the Intelli Scan 1600 (Quanto technology) and the SilverFast Ai program or FUSION FX chemiluminescence detection system (Peqlab).

### Chromatin immunoprecipitation coupled to quantitative PCR (ChIP-qPCR)

The ChIP-qPCR was performed as previously described [39]. In brief, cells were grown to late logarithmic phase (OD_600_ = 1.2) in 50 ml cultures. For crosslinking, 1.39 ml formaldehyde was added and incubated at room temperature for 10 min. The culture was mixed regularly by swirling. To stop crosslinking, 2.75 ml of 2.5 M glycine was added to the cells for 5 min at room temperature. Cells were pelleted for 10 min at 4000 xg and 4 °C, washed with 20 ml cold TBS (50 mM Tris pH 7.4, 150 mM NaCl)/125 mM glycine, washed with 20 ml cold TBS, resuspended in 1 ml TBS, transferred to a 2—ml screw top tube, pelleted, frozen in liquid nitrogen and stored at −20 °C. For lysis, cells were resuspended in 400 µl ChIP lysis buffer (50 mM HEPES pH 7.5, 140 mM NaCl, 1% Triton, 0.1% sodium deoxycholate, 1 mM EDTA), 20 µl protease inhibitor added and an equal amount of glass beads. The Fast Prep was used three times for 40 s at 4.5 m/s, with 5 min incubation on ice in between steps. Lysed cells were centrifuged at 4 krpm and 4 °C for 5 min. The pellet was emulsified and chromatin shredded using a water bath sonicator for 2.5 min. Cell debris were removed by centrifugation at 13 krpm for 10 min at 4 °C and the protein concentration of the supernatant was measured. To verify efficient shearing of DNA, 20 µl of the lysate was treated with 1 µl 10% SDS and 0.5 µl Proteinase K for 1 h at 37 °C, and crosslinking was reverted by incubation for 2 h at 65 °C. The DNA was isolated using Phenol–Chloroform extraction, and the pellet was resuspended in 20 µl TE pH 8.0 and treated with 40 µl/ml RNase for 1 h at 37 °C before analysis by agarose gel electrophoresis. When fragment sizes ranged from 100 to 1000 bp, the material was used for further steps. As a protein lysate control for SDS-PAGE, 20 µl lysate were mixed with 2 × sample buffer (125 mM Tris pH 6.8, 2% SDS, 10% glycerol, 5% 2-mercaptoethanol, bromphenol blue). For a DNA lysate control, 2 µl of lysate was mixed with 150 µl ChIP elution buffer (50 mM TRIS pH8.0, 10 mM EDTA, 1% SDS). 10 mg protein were diluted in 400 µl ChIP lysis buffer and incubated end-over-end with 10 µl GFP Selector beads (NanoTag) for one hour at 4 °C. Beads were washed twice with ChIP lysis buffer with protease inhibitors (0.2 mM PMSF, 500 µM DTT, 1:1000 Protease Inhibitor), washed twice with High salt buffer (50 mM HEPES pH7.5, 500 mM NaCl, 1% Triton, 0.1% sodium deoxycholate, 1 mM EDTA, 0.2 mM PMSF, 500 µM DTT, 1:1000 Protease Inhibitor), washed twice with wash buffer (10 mM TRIS pH 8.0, 250 mM LiCl, 0.5% NP-40, 0.5% sodium deoxycholate, 1 mM EDTA) and twice with TE buffer pH 8.0. Then, the beads were resuspended in 170 µl ChIP elution buffer, incubated at 65 °C with 950 rpm, centrifuged briefly and the supernatant transferred to a fresh tube. For SDS-PAGE, 20 µl were retained. Crosslinks were reversed by incubation of lysate and eluate at 65 °C over-night. The DNA was purified using the NucleoSpin Gel and PCR Clean-up Kit (Macherey Nagel) according to the manufacturer’s instructions and eluted in 50 µl nuclease-free water. Analysis was performed by qPCR (see below).

**Western blot analyses and detection.** All antibodies used in this study are listed in Supplementary Table S9. Secondary anti-mouse IgG-HRP and anti-rabbit IgG-HRP (Dianova) were detected with WesternBright Chemilumineszenz Substrat Quantum (Biozym) and detected with a FUSION-SL or FUSION FX chemiluminescence detection system (Peqlab). Alternatively, secondary anti-rabbit IRDye680 or IRDye800CW diluted 1: 10,000 were used for detection with a LI-COR Odyssey scanner and images were processed in Image Studio 5.2.5 (LI-COR). Western blot signals were quantified with the Adobe Photoshop 2021 or the Fiji-software. The signal intensity of the co-precipitated protein bands was related to the intensity of the bait protein bands. The ratio between mutant strains was compared to wild type.

### Co-immunoprecipitation (Co-IP)

To analyze the interaction of a specific protein with other proteins, Co-IP was carried out as described before [40]. For this, 400 ml yeast cultures were grown to exponential phase and harvested by centrifugation for 5 min at 4 °C and 4 krpm. Cells were resuspended in ddH_2_O, pelleted by centrifugation and frozen in liquid nitrogen. The cells were lysed by adding one cell-pellet volume of glass beads and PBSKMT buffer (1 × PBS (137 mM NaCl, 3 mM KCl, 10 mM NaH_2_SO_4_, 2 mM KH_2_SO_4_), 3 mM KCl, 2.5 mM MgCl_2_, 0.5% Triton), as well as 50 μl/ml Protease inhibitor to the pellet. The mix was vortexed three times for 30 s at 5.5 rpm/sec in the Fast Prep machine. Between vortexing steps, the samples were cooled down on ice for 5 min. The lysate was centrifuged for 5 min at 4 krpm and 4 °C, transferred to a 1.5 ml tube and centrifuged for 15 min at 13 krpm and 4 °C. This step was repeated until the lysate appeared clear. As lysate protein controls, 20 μl per sample were taken. The remaining lysate was divided in two and diluted with the same volume PBSKM without Triton but with Protease Inhibitor to reduce the detergent concentration. One sample was treated with 200 μg/ml RNase A and all samples were incubated at 25 °C for 30 min. While lysing cells, 10 μl GFP-Selector beads, 10 μl MYC-Trap beads (ChromoTek), 20 μl Protein G Sepharose beads (GE Healthcare) or 20 μl IgG-Sepharose beads (GE Healthcare) (for TAP-IPs) per reaction were prepared by washing them twice with PBSKMT, blocking them with 1 ml of 10 mg/ml BSA and 10 μl glycogen (20 mg/ml) per reaction for 30 min at 4 °C with end-over-end rotation. Beads were washed three times with PBSKMT. After RNase treatment, the lysate was incubated with 10 μl beads per reaction for 1 h at 4—8 °C on an end-over-end rotator. When Protein G Sepharose beads and IgG-Sepharose beads were used, the incubation time was increased to 3 and 4 h respectively. Beads were centrifuged for 1 min at 4 krpm (2 min 2 krpm for IgG-Sepharose beads) and carefully washed with 1 ml PBSKMT buffer five to ten times. After removing the supernatant for the last time, 35 μl 2 × sample buffer was added to each sample and incubated for 5 min at 95 °C. The bait and co-precipitated proteins were analyzed via western blotting.

### Total RNA isolation

Total RNA isolation was carried out with the NucleoSpin RNA Kit from Macherey–Nagel. All steps were conducted according to the manufacturer's description. Alternatively, cell pellets were lysed in TRI reagent (Sigma-Aldrich) by vortexing with zirconia/silica beads (BioPsec Products) at 4 °C. RNA extraction was then performed according to the TRI reagent manufacturer’s instructions. Purified RNAs were measured using a NanoDrop UV/Vis Spectral photometer (Peqlab) or a Nanodrop One^c^ (ThermoFisher). A defined amount of RNA was reverse transcribed with FastGene Scriptase II (Nippon Genetics) for subsequent qPCR analyses, or RNA was directly used for northern blot analysis.

### Northern blot analysis

Northern blotting was carried out as described before [41]. 50 ml yeast cultures were harvested in logarithmic phase and RNA was isolated. A 1.2% agarose gel was loaded with 2—5 µg RNA that had been mixed with 20 µl Glyoxal Mix (6 ml DMSO, 2 ml deionized glyoxal, 1.2 ml 10 × BPTE, 600 µl 80% Glycerol) and incubated for 1 h at 55 °C. As a running buffer 1 × BPTE (10 mM PIPES, 30 mM Bis–Tris, 1 mM EDTA) was used. After appropriate separation, the gel was rinsed for 10 min with DEPC-treated water, soaked for 20 min in 75 mM NaOH with gentle shaking for denaturation, rinsed for 5 min with DEPC-treated water and soaked twice for 15 min in 0.5 M Tris–HCl pH 7.4/1.5 M NaCl for neutralization. After another rinse in DEPC treated water, the gel was soaked for 20 min in 6 or 10 × SSC (1.5 M NaCl, 150 mM sodium citrate, pH 7.0) and RNA was transferred to positively charged nylon membrane (Amersham Hybond -N^+^ (GE Healthcare) over night by capillary blotting or in 2 h using a vacuum blotting system. RNA was crosslinked to the membrane (7 min 5000 J/cm^2^) and the membrane baked at 80 °C for two hours. Subsequent hybridization was done with a DIG-labelled probe and detection occurred through using the anti-Digoxigenin-alkaline phosphatase (1:10,000, Roche). As a substrate, CSPD (1:100 in the detection buffer) was used. Images were taken with the FUSION-SL or FUSION FX chemiluminescence detection system (Peqlab). Alternatively, membranes were incubated with pre-hybridization buffer (SES1; 250 mM NaPi pH 7.4 (NaH_2_PO_4_.H_2_O + Na_2_HPO_4_), 7% SDS and 1 mM EDTA) before addition of a 5’ [^32^P]-labelled antisense DNA oligonucleotide (Supplementary Table 8) over night at 37 °C. Radiolabelled probes were generated using T4 PNK. Membranes were then washed at 37 °C for 30 min each in 6 × SSC and 2 × SSC + 0.1% SDS. Membranes were exposed to phosphorimager screens and RNA signals detected using a Typhoon FLA 9500.

### DIG-labeling probe preparation

To generate sequence-specific DIG-labeled RNA probes, PCR was done with primers specific for amplification of a 300 bp fragment of the sequence of interest. The reverse primer contains the T7 start site. A digoxigenin (Dig)-labeled probe was prepared by in vitro transcription using DIG DNA Labeling Mix (Roche) and T7 Polymerase (Thermo Fisher Scientific). RNA was precipitated with ethanol and 4 M LiCl. After washing with 70% ethanol RNA was dissolved in 20 µl 1 × TE buffer, 20 µl of deionized formamide and 60 µl HybMix (50% (v/v) deionized formamide, 5 × SSC, 1 × Denharts solution, 0.1 mg/ml Heparin) were added and the probes stored at −20 °C.

### RNA-seq

RNA-seq analysis was conducted at the Next Generation Sequencing (NGS) Core Facility for Integrative Genomics (NIG), University Medical Center Göttingen. A modified TruSeq Stranded Total RNA Library Prep (Cat. No. 20020596) was used without rRNA depletion, starting with 200 ng of total RNA. The RNA sample quality and subsequent library quality were verified using a Fragment Analyzer. A NovaSeq6000 was used for sequencing (PE 100 cycles; 30 Mio reads/sample, 50 bp). Hisat2 was used to map.fastq files to the *S. cerevisiae* genome sacCer3 (Kim, Langmead, and Salzberg 2015). For abundance measurement of reads overlapping with the 5’ ETS1, ITS1, ITS2, 3’ *ETS* and 18S rRNA, 25S and 35S rRNA (sum of all other counts), featureCounts was applied [42]. Subsequently, counts in *npl3∆* were related to the wild type counts. For the coverage analysis, Hisat2 was run twice to generate strand-specific.bam files. Once, the forward strand was skipped and once, the reverse strand was skipped. Next, the fragment coverage (defined see table A, B in Source data 2) of the reverse strand was calculated with deeptools2 and a bin size of 1 [43] to get the fragment coverage of the 35S rRNA. To compare the mutant with the wild type, the logarithm base 2 of the quotient between *npl3∆* and the wild type was calculated.

### RNA co-immunoprecipitation (RIP) experiments without and with crosslinking

The experiments were essentially conducted as published earlier [6]. All yeast strains were grown to mid log phase (2 × 10^7^ cells/ml) at 25 °C and harvested. Where indicated, UV light (twice for 3.5 min with 254 nm) was used to crosslink proteins with RNA in cells prior to harvesting. Cells were lysed in 1 pellet volume RIP buffer (25 mM Tris–HCl, pH 7.5, 150 mM NaCl, 2 mM MgCl_2_, 0.2% (v/v) Triton X-100, 0.2 mM PMSF, 0.5 mM DTT, 10 U RiboLock™ RNase Inhibitor (Thermo Scientific)) and 1 volume of glass beads. Cells were lysed by vigorous vortexing three times for 30 s at 5.5 m/s using the FastPrep®−24 instrument (MP Biomedicals). Input samples were taken for protein detection by western blotting and RNA isolation using Trizol-chloroform (Ambion® RNA by Life technologies ™). Co-immunoprecipitation experiments were carried out at 4 °C by incubating the lysates with Myc-Trap beads or GFP-selector beads for 1 h. Afterwards, the beads were washed five times with RIP buffer, and two times with Proteinase K buffer (50 mM Tris pH7.5, 50 mM NaCl, 0.2% Triton, 0.5 mM DTT), and split into two portions after the last washing step. Proteins were detected by western blotting. RNA samples were incubated with 5 μl DNaseI (Roche) at 25 °C for 1 h. In case of prior UV crosslinking, the RNA samples were treated with proteinase K (by addition of 0,5% SDS, 5 mM EDTA and 40—80 µg Proteinase K) at 55 °C for 1 h. The RNA was purified via Trizol–chloroform extraction. In an additional DNA digestion step, Turbo DNAse (Thermo Fisher Scientific) was used according to the manufacturer’s instructions. Another RNA precipitation via adding 0.1 volume 3 M sodium acetate, pH 5.2, 2.5 volumes of 99% pure ethanol and 1 μl Glycoblue followed at −20 °C over night. Equal amounts of RNA purified from lysate, which reflects the input control, or eluate RNA were finally used as template for reverse transcription by FastGene Scriptase II (Nippon Genetics) for subsequent qPCR analyses. RNA levels in the eluates were measured by RT-qPCR and normalized to mitochondrial 21S rRNA or mitochondrial mRNA *QRI7*. All samples were related to the no tag control.

#### 3’- RACE

The experiments were, in principle, conducted as described earlier [44]. All yeast strains were grown to mid log phase (2 × 10^7^ cells/ml) at 25 °C and the RNA was isolated from 10 ml cultures. Reverse transcription was performed using an oligo d(T_17_) primer containing a unique adapter sequence at its 5’end and either a C, G or A at its 3’end, to prevent internal binding of the poly(A)-tail. The generated cDNA was subsequently amplified using the qPCRBIO SyGreen Mix ROX from Nippon Genetics and a primer binding upstream of the A_2_ cleavage site in the 35S rRNA (HK3492) together with a reverse primer that hybridizes to the adapter sequence. PCR products were separated on 2% agarose gels and specific bands were cut and purified from the gel using the NucleoSpin Gel and PCR Clean-up Kit from Macherey–Nagel and subsequently sent for Sanger sequencing.

### Quantification

All experiments shown in this work were repeated at least three times independently. Quantifications of western blots, northern blots or qPCR experiments were analyzed for significance using a Student's two-tailed, two-sample, equal variance *t*-test. Error bars represent the standard deviation. P-values are indicated as follows: ***P < 0.001, **P < 0.01, *P < 0.05.

## Results

### Npl3 is recruited co-transcriptionally to RNAPI transcripts and associates with pre-ribosomal complexes

**Fig. 1:**
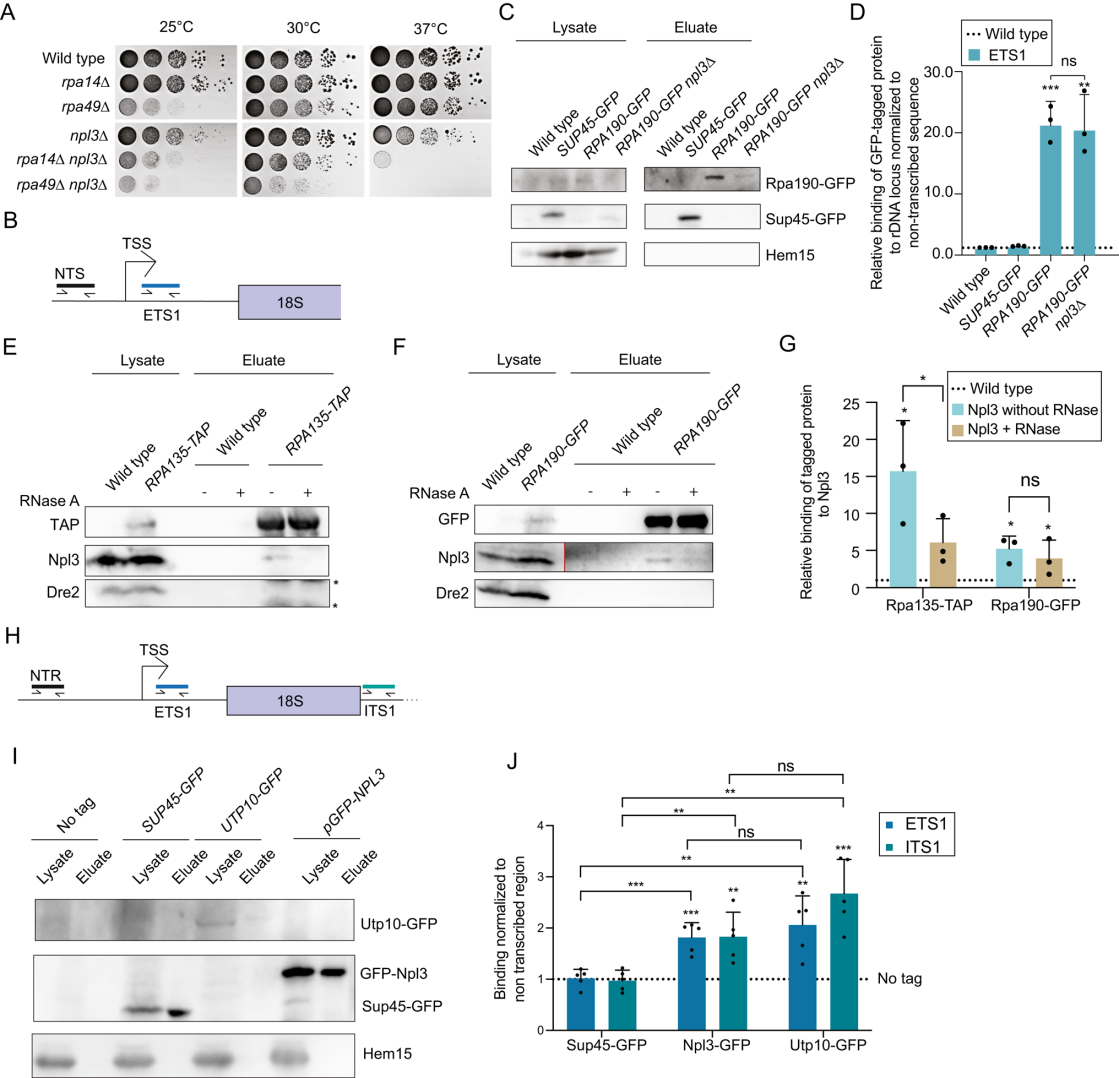
Npl3 binds co-transcriptionally to pre-rRNA**.** (**A**) Deletion of *NPL3* leads to a growth defect or synthetic lethality with deletions of genes encoding RNAPI proteins. Ten-fold serial dilutions of the indicated strains were spotted onto YPD plates that were incubated at the indicated temperatures for three days. n = 3. (**B**) Primer pairs used for the ChIP-qPCR analysis shown in C and D. (**C**) Western blot analysis of the ChIP-qPCR samples validates the protein pulldown. GFP-tagged proteins in the lysate and eluate of the indicated strains are shown. The mitochondrial protein Hem15 was used as a negative control. The lysate and eluate of Sup45-GFP were detected using different exposure times (lysate 6 and eluate 15 s). (**D**) Binding of GFP-tagged Rpa190 to the rDNA locus does not change when *NPL3* is deleted. Binding of Rpa190-GFP to the ETS1 was analyzed via qPCR, related to no tag control and normalized to the non-transcribed sequence (NTS). n = 3. *P < 0.05; **P < 0.01; ***P < 0.001. (**E**–**G**) Western blot analysis of co-IPs with TAP-tagged Rpa135 (**E**) or GFP-tagged Rpa190 (**F**) and Npl3 are shown. The mitochondrial protein Dre2 served as negative control. * are degradation products of Rpa135-TAP. The vertical red line shows where the left and the right side of the membrane were separated to detect the proteins with different exposure times (lysate 5 and eluate 20 s). (**G**) Npl3 interaction with RNAPI is RNA-sensitive. Quantification of the interaction between Rpa190 and Rpa135 and Npl3 is shown. n = 3. (**H**) Primer pairs used for analysis of ChIP experiment shown in I and J. (**I**) Pulldown of GFP-tagged proteins was validated via western blot analysis. Hem15 served as a negative control. * originates from the GFP-Npl3 detection. (**J**) Npl3 is recruited co-transcriptionally to the rDNA locus. Binding of GFP-tagged proteins to ETS1 and ITS1 compared to the no tag control is shown. Utp10 and Sup45 served as a positive and negative controls, respectively. n = 5

Co-transcriptional recruitment of Npl3 to RNAPII transcripts has been shown earlier [45], but Npl3 not only binds to mRNAs but also to rRNA [9, 12] (Fig. S1A). We therefore investigated potential interactions of Npl3 with RNAPI and the rDNA because such interactions could indicate co-transcriptional association with pre-rRNAs. First, potential genetic interactions between *NPL3* and two non-essential components of the RNAPI were investigated. All yeast strains used in this study are in the *BY4741* background in which the deletion of *NPL3* is viable but has a mild growth defect at 25 °C [6]. An *npl3∆* strain was crossed with *rpa14∆* and *rpr49∆* strains and growth of the double mutants was analyzed. Genetic interactions of Npl3 with both RNAPI mutants were observed (Fig. 1A), connecting the function of Npl3 to the RNAPI-mediated rDNA transcription and/or synthesis of the pre-rRNA transcript. Next, to determine whether Npl3 plays a role in the recruitment of RNAPI to the rDNA, association of the largest RNAPI subunit Rpa190 with rDNA was analyzed by ChIP-qPCR experiments in wild type and *npl3∆* cells. As a negative control to demonstrate the specificity of rDNA interaction, the cytoplasmic translation termination factor Sup45 was included. The ChIP-qPCR was conducted with primers that amplify part of the 5’ *ETS* or a non-transcribed sequence (*NTS*) of the rDNA locus (Fig. 1B). Across the replicate experiments, no strong differences in the amount of 5’ ETS-encoding rDNA recovered with Rpa190, in the wild type and *npl3∆* strains were observed (Fig. 1C, D, Source data 1A), suggesting that the absence of Npl3 does not prevent the interaction of RNAPI with the rDNA promoter. The slight reduction of the Rpa190 protein band in the western blot might rather be due to the slower growing *npl3*∆ mutant. Importantly, the DNA binding of Rpa190 in wild type and *npl3∆* were comparable (Fig. 1C). The interaction of Npl3 with RNAPI may therefore rather reflect a role of RNAPI in loading Npl3 onto nascent pre-rRNAs. This would be in line with the presence of tandem RNA-binding domains in Npl3 and its known association with (pre-)rRNAs [9, 12]. To investigate a potential physical interaction of Npl3 with RNAPI, we carried out co-immunoprecipitation (co-IP) experiments with Rpa190 and Rpa135 and found that small amounts of Npl3 are recovered with both proteins (Fig. 1E-G, Source data 1B, C). While the interaction of Npl3 with Rpa135 was RNase sensitive, the interaction with Rpa190 was reduced but not eliminated upon RNase A treatment, suggesting a potential direct contact between Npl3 and Rpa190. This physical interaction of Npl3 with RNAPI suggests the association of Npl3 with chromatin containing the rDNA locus, and this was verified using ChIP-qPCR experiments. Again, the cytoplasmic translation factor Sup45 served as a negative control in this experiment. Although the pull down for Utp10 was overall lower, we found that Npl3 shows a comparable binding to the ETS1 and ITS1 rDNA, yet milder than Utp10 to the ITS. Utp10 is a protein of the SSU processome that is recruited co-transcriptionally to nascent pre-ribosomal particles (Fig. 1H-J, Fig. S1B, Source data 1D), demonstrating that Npl3 associates with the rDNA. It has previously been shown that Npl3 associates with the 35S pre-rRNA and this was confirmed using RNA co-IP experiments coupled to RT-qPCR [12]. Using a primer pair targeting the 5’ *ETS*, a robust association of Npl3 with the nascent pre-rRNA transcript was observed. Thus, we propose that Npl3 is loaded co-transcriptionally onto pre-rRNA. To investigate if Npl3 also associates with pre-ribosomal complexes, cellular extracts prepared in the absence of cycloheximide (polysome run off) were separated by sucrose density gradient centrifugation. Monitoring absorbance at 254 nm enabled fractions containing (pre-)40S, (pre-)60S and 80S/SSU processome complexes to be identified and these migration patterns were confirmed by western blotting for ribosomal proteins of the SSU (Rps14) and LSU (Rpl3) (Fig. S1C, D). Analysis of the distribution of Npl3 showed that much of the protein is present in fractions containing free proteins and non-ribosomal complexes. However, a portion (approximately 5%) of Npl3 also co-migrates with pre-40S, pre-60S and soluble SSU processome particles (Fig. S1D), supporting a role for this protein during ribosomal subunit assembly. While the portion of Npl3 co-migrating with pre-ribosomal subunits is low, this is in line with the multifunctionality of Npl3 and its prominent role as an mRNA surveillance factor. Supporting the specificity of the association of this portion of Npl3 with pre-ribosomes, the cytoplasmic kinase Pgk1 is not detected in fractions containing pre-ribosomal complexes (Fig. S1D). Together, these findings show that Npl3 is loaded onto nascent pre-rRNAs co-transcriptionally by RNAPI and remains associated to soluble SSU processome particles.

### *The ETS1 fragment (5’-A*_*0*_*) and the aberrant 23S pre-rRNA accumulate in npl3∆*

**Fig. 2:**
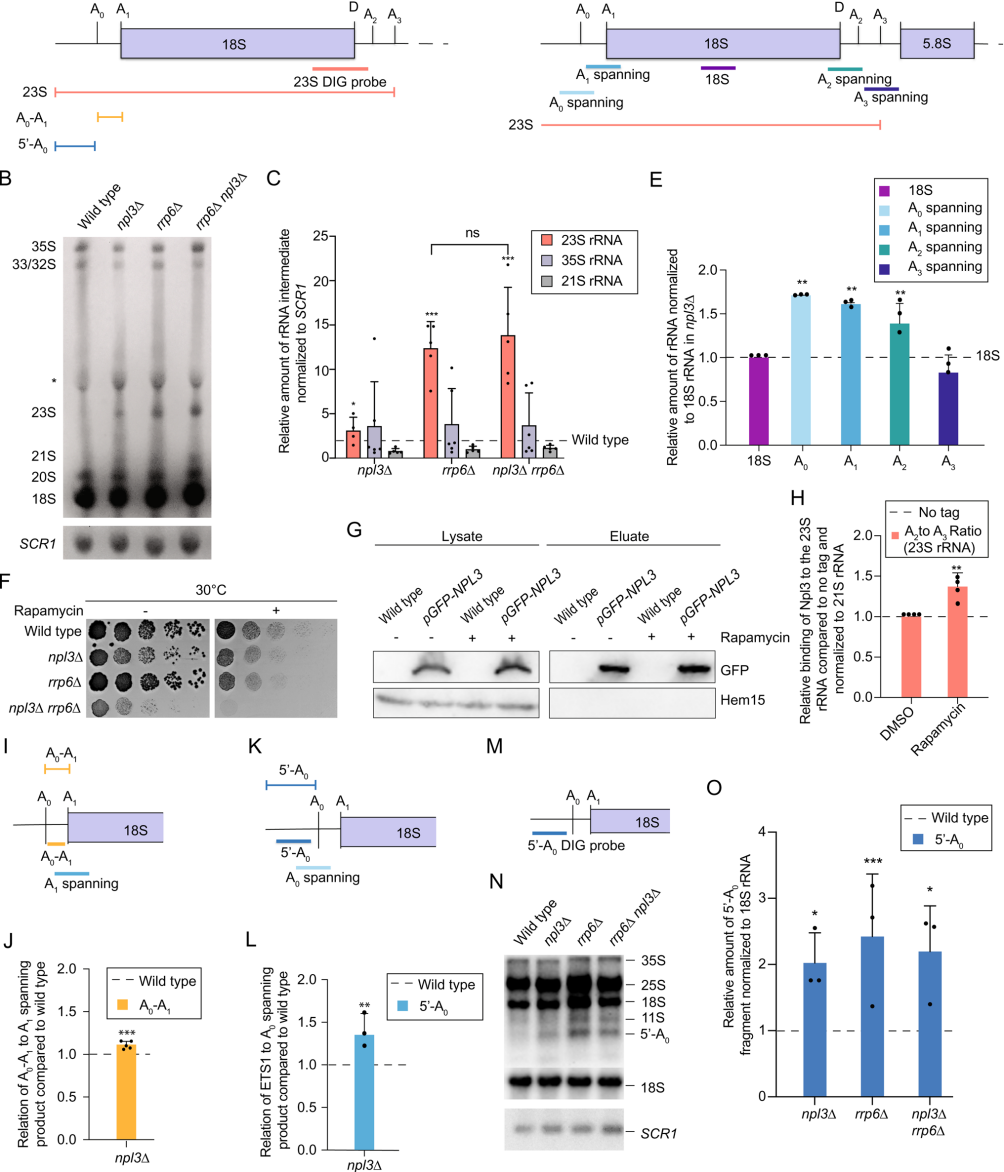
Deletion of *NPL3* leads to an accumulation of faulty pre-rRNA fragments**.** (**A**) The digoxygenin (DIG)-labelled probe used for the detection of pre-rRNA fragments by northern blotting is indicated in orange on a scheme of the 5’ end of the pre-rRNA transcript. The excised spacer fragments 5’-A_0_ (blue) and A_0_-A_1_ (yellow) are shown as well as the aberrant 23S pre-rRNA (orange). (**B**) Northern blotting using the DIG-labelled probes and alkaline phosphatase coupled anti-DIG antibody detecting the indicated pre-rRNA species in the indicated strains. Detection of the RNAP III transcript *SCR1* served as a loading control. * indicates cross-reaction of the DIG-labelled probe with the 28S rRNA. Five independent experiments were preformed and a representative image is shown. (**C**) Quantification of the 23S*,* 35S and 21S pre-rRNA levels relative to *SCR1* in northern blots as in (**B**). (**D**) The regions amplified by qPCR using primer pairs spanning specific pre-rRNA cleavages sites (A_0_, A_1,_ A_2,_ A_3_) are shown on a scheme of the 5’ end of the pre-rRNA transcript. (**E**) The 23S pre-rRNA accumulates in *npl3∆*. qPCR analysis of pre-rRNAs from wild type and *npl3∆* cells using the indicated primers pairs is shown. n = 5. (**F**) Deletion of *NPL3* and *RRP6* is lethal at 30 °C upon rapamycin treatment. Ten-fold serial dilutions of the indicated strains were spotted onto YPD plates containing either DMSO or rapamycin (200 ng/ml) that were incubated at 30 °C for 3 days. n = 3. (**G**, **H**) The Npl3 binding to the 23S rRNA increases upon rapamycin treatment. Cells were grown to log-phase and treated with either DMSO or rapamycin (200 ng/ml) for 1 h. RIP of GFP-tagged Npl3 was conducted. Western blot analysis (**G**) verifies the pull down and Hem15 served as negative control. (H) The ratio of A_2_ to A_3_ spanning (23S rRNA) qPCR results are shown n = 4. *P < 0.05; **P < 0.01; ***P < 0.001. (**I**) qPCR products detecting uncleaved A_1_ fragments and the A_0_-A_1_ region are indicated. (**J**) The A_0_-A_1_ fragment is increased in the *NPL3* deletion strain. qPCR detection to distinguish between A_0_-A_1_ and the 23S or 35S pre-rRNAs is depicted. Relation of the amount of the A_0_-A_1_ fragment to the A_1_ products. n = 5. (K) qPCR products detecting uncleaved A_0_ products and the ETS1 region are indicated. (**L**) 5 ‘-A_0_ fragments accumulate in *npl3∆*. Relation of the amount of the 5 ‘-A_0_ fragment to the 23S and 35S rRNA products. n = 7. (M) Digoxygenin (DIG)-labelled probe used for the detection of ETS1 fragment 5’-A_0_ in northern blots is depicted in blue. (**N**) Northern blot analysis with an ETS1-DIG-labelled probe detects an accumulation in *npl3∆*. The *RRP6* deletion strain served as positive control for 5’-A_0_ fragment accumulation. *SCR1* and 18S rRNA served as loading controls. n = 3. (**O**) Quantification of three independent northern blots, one of which is shown in (**O**). *P < 0.05; **P < 0.01; ***P < 0.001

Accumulation of the 23S pre-rRNA upon Npl3 depletion via a repressible promoter and upon deletion of the nuclear exosome component *RRP6* have been shown earlier [26, 33, 36]. We affirmed that 23S pre-rRNA also accumulates in the *NPL3* knock out strain and investigated whether the double mutant *npl3∆ rrp6∆* accumulates additional 23S pre-rRNA, which was not the case as shown in northern blot analysis (Fig. 2A-C). The appearance of the 23S rRNA in *npl3∆* suggests that this guard protein might be involved in pre-rRNA surveillance. As the level of the 23S pre-rRNA did not significantly increase when comparing *npl3*∆ *rrp6*∆ with the single mutant *rrp6∆*, these proteins likely act in the same degradation pathway. Moreover, as the 35S pre-rRNA level was not significantly reduced in *npl3∆*, synthesis of the pre-rRNA appears largely unaffected by the absence of Npl3 very similar to the Npl3/Nop3 depletion experiments shown earlier [36]. These findings were confirmed with qPCRs that amplified the A_0_, A_1_, A_2_ and A_3_ spanning regions (Fig. 2D). While the products spanning A_0_, A_1_ and A_2_ were significantly increased, the A_3_ spanning product decreased in *npl3∆* (Fig. 2E), which supports the accumulation of the 23S pre-rRNA. An increased level of the 23S pre-rRNA is also detectable upon the addition of rapamycin or TBB to wild type cells (Fig. S2A) as these drugs promote switching from a productive (A_2_) to a non-productive (A_3_) pre-rRNA processing pathway [46]. As rapamycin had a stronger effect on 23S rRNA production we investigated whether cells that lack Npl3 would be hypersensitive to this drug. Moreover, we addressed whether increasing amounts of the 23S rRNA in cells could be toxic. Thus, we compared the growth of the four strains used above in the presence of rapamycin and detected that its combination with all strains further inhibited their growth. Strikingly, growth of the double mutant *npl3∆ rrp6∆* was not possible in the presence of rapamycin (Fig. 2F and Fig. S2B-D), suggesting that increased amounts of the 23S dead end-product overwhelm pre-rRNA surveillance and elimination overburdens cells. Importantly, Npl3 binding to the 23S pre-rRNA was increased upon rapamycin treatment (Fig. 2G, H), suggesting a direct role of Npl3 in removal of the dead-end 23S rRNA.

Both, the northern blotting and qPCR approaches indicated no significant change in 35S pre-rRNA level in *npl3∆*, but elevated 23S pre-rRNA levels in *npl3*∆ (Fig. 2B, C, E), which confirms the previous finding of pulse chase experiments [36]. This qPCR approach cannot, however, unambiguously determine whether the excised spacer fragments generated through the A_0_ and A_1_ cleavages in the ETS1 region (5’ to A_0_ and A_0_ to A_1_) are less efficiently degraded in *npl3*∆ than in wild type. Therefore, to monitor the levels of these fragments, the A_0_-A_1_ region was specifically amplified (Fig. 2I) and related to the A_1_ spanning region. This revealed a slight but significant accumulation of the A_0_-A_1_ in *npl3∆* (Fig. 2J). An even stronger accumulation of the 5’-A_0_ fragment was observed in *npl3∆* (Fig. 2K). Amplification of this region and relation to the A_0_ spanning region demonstrated a ~ 1,threefold higher amount of the 5’-A_0_ product in *npl3∆* compared to wild type (Fig. 2L). In line with the qPCR results, a ~ twofold accumulation of the 5’ ETS1 fragment (5’-A_0_) was also detected in *npl3∆* by northern blotting (Fig. 2M-O). This is similar to the accumulation of this fragment in *rrp6∆* and the additional loss of both Rrp6 and Npl3 did not further exacerbate this defect (Fig. 2M-O). These results suggests that Npl3 is required for efficient turnover of excised fragments of the 5’ ETS during the early stages of ribosome assembly.

### Assembly of the SSU processome is perturbed in npl3∆

**Fig. 3:**
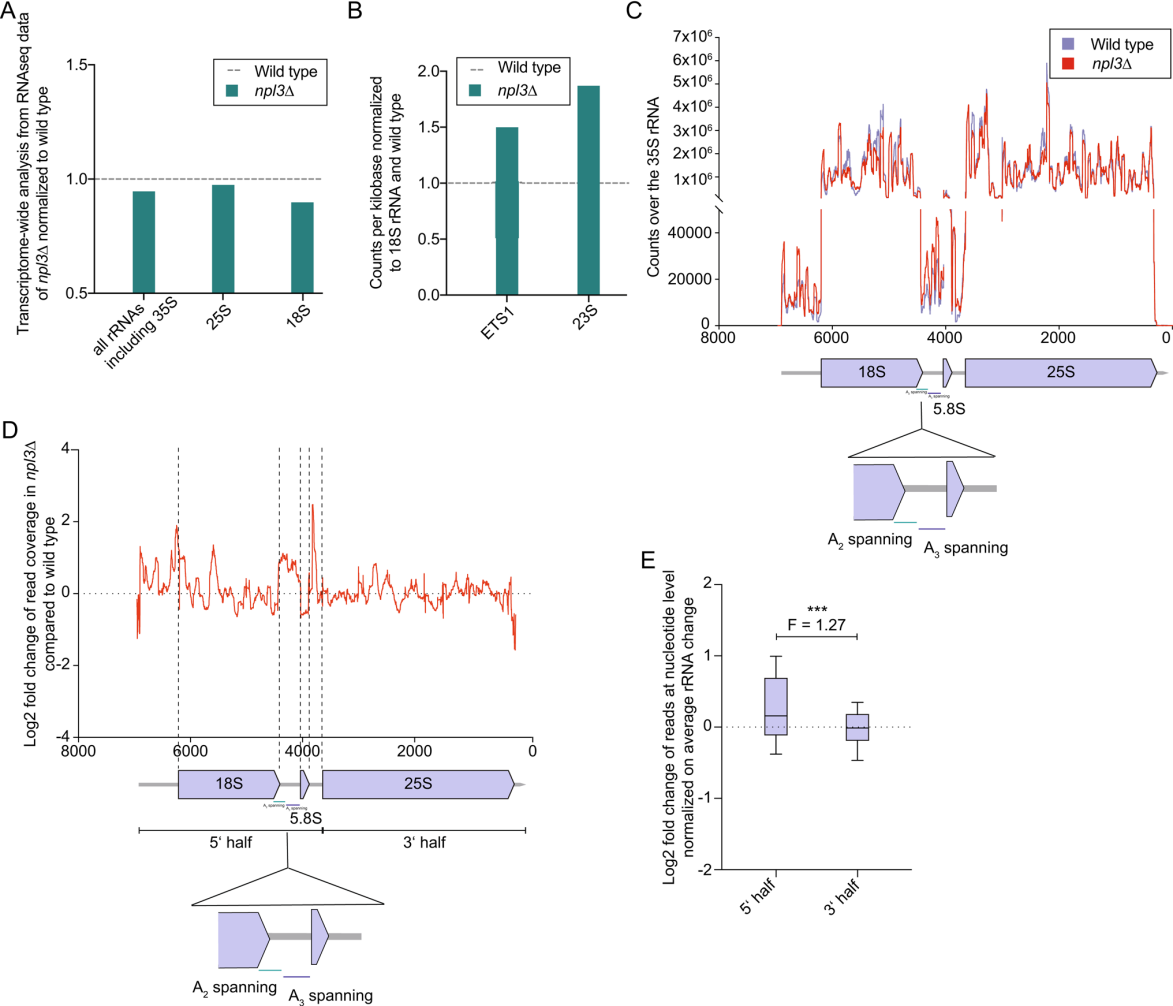
The levels of the 18S rRNA and the SSU pre-rRNA regions are altered when *NPL3* is deleted**.** (**A**-**E**) Analysis of the RNA-seq data of wild type and *npl3∆* cells that was conducted without rRNA depletion. (**A**) RNA-seq reveals altered (pre-)rRNA amounts in *npl3∆.* The sequencing reads mapping to different (pre-) rRNA regions in wild type and *npl3∆* were determined and related to each other. (**B**) RNA-seq reveals an accumulation of reads mapping to the ETS1 region as well as to the 23S rRNA (and 35S rRNA) spanning region in *npl3∆.* For normalization, the reads per kilobase were compared to the 18S rRNA. (**C**) The read coverage over the 35S *pre-*rRNA in *npl3∆* and wild type was compared and the changes in *npl3*∆ are depicted. (**D**) Especially the 5’ half of the pre-rRNA is altered in *npl3∆*. The Log2 fold change of the read coverage in *npl3∆* compared to wild type was calculated. The vertical dotted lines indicate the start and/or end of the 18S, 5.8S and 25S rRNA. (**E**) A summary of the changed reads shown in (**D**) is depicted. The nucleotides of the rRNA were divided into a 5' and 3' part (**D**) and their log2 fold change in read coverage is shown as a box plot (10th to 90th percentiles). An F-test was used to test for a difference in the magnitude of variance. *P < 0.05; **P < 0.01; ***P < 0.001

To date, no RNA-seq data are available for *npl3∆*. To obtain an overview of (pre-)rRNA levels, total RNA from *npl3∆* cells was subjected to RNA-seq without prior rRNA depletion, which is often applied during RNA-seq analyses. As expected, low read numbers derived from mRNAs were obtained but a clear profile of reads mapping to the (pre-) rRNA was obtained. RNA-seq data revealed an overall slight reduction of the (pre-)rRNAs in *npl3∆* compared to wild type (Fig. 3A). Importantly, while lack of Npl3 only mildly reduced the number of sequencing reads mapped to the 25S rRNA sequence, markedly less sequencing reads were derived from the 18S rRNA in the *npl3∆* strain compared to wild type (Fig. 3A). Although Npl3 is already known to be important for the export of the large ribosomal subunit and subsequently for ribosomal subunit joining, (12,47), the sequencing result suggests that Npl3 might in fact more strongly impact the maturation of the small ribosomal subunit than the large subunit. Moreover, counting A_2_-spanning reads that can derive from the 23S and 35S pre-rRNAs revealed an increase (Fig. 3B). However, as the 35S pre-rRNA is not elevated in *npl3∆* (Fig. 2A), this increase can be attributed to an accumulation of the faulty 23S pre-rRNA. Likewise, the increase in the number of reads mapping to the 5’ ETS1 in *npl3∆* is consistent with the accumulation of both the 23S pre-rRNA and the 5’-A_0_ and A_0_-A_1_ fragments (Fig. 2A, J-P; Fig. 3B).

Interestingly, when analyzing the read-coverage in the *npl3∆* strain and compared to that of wild type, it became apparent that it is elevated in some regions and reduced in others (Fig. 3C, D). One possible reason for the distinct read-coverage is a potentially altered secondary structure of the pre-rRNA when Npl3 is missing. Strong secondary structures can affect RNA fragmentation and the subsequent cDNA synthesis during the RNA-seq library preparation [47]. This is presumably the reason why we see differences in the log2 fold change of the reads mapping to each nucleotide of the pre-rRNA transcript in *npl3∆* compared to wild type. Deletion of *NPL3* results not only in a significantly higher level of sequences for the 18S rRNA, but to all sequences derived from the 5’ half of the 35S rRNA (including the 5’ ETS, 18S, 5,8S and the ITS1 and ITS2), compared to the 3’ located half without the 5’ ETS and the ITS*s* but including the 25S rRNA and the 3’ ETS (Fig. 3D, E). This indicates that the deletion of *NPL3* mainly affects SSU maturation and the removal of the 5’ ETS, ITS1 and ITS2 spacer regions. Correct assembly of the SSU processome is a pre-requisite for pre-rRNA events necessary for the formation of the 18S rRNA [48, 49].

### Npl3 shows physical and genetic interactions with SSU processome components

**Fig. 4:**
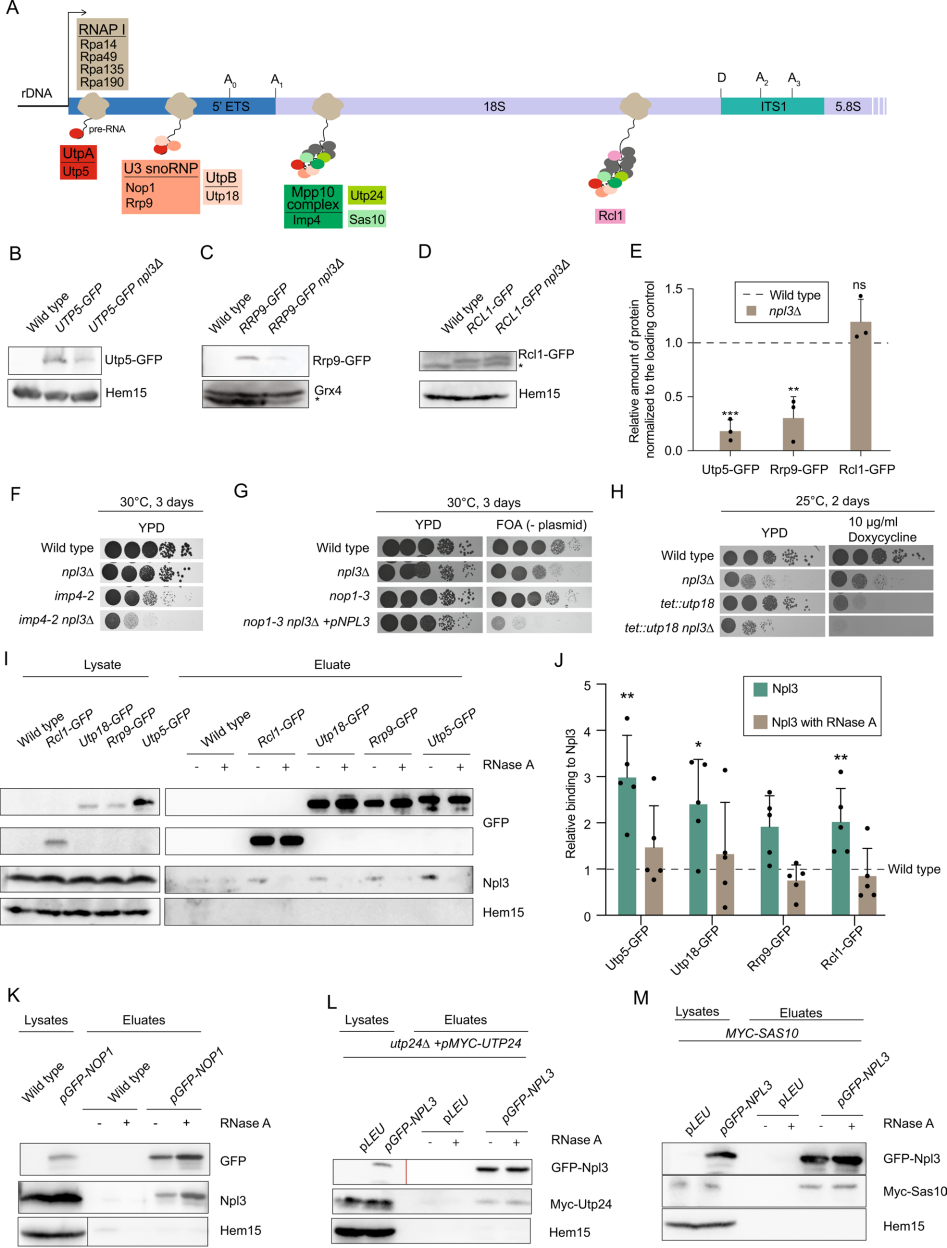
Npl3 interacts with the SSU processome and is important for its assembly on the pre-rRNA**.** (**A**) A simplified scheme of the SSU processome assembly pathway highlights the factors that were investigated in this study. (**B**-**D**) Western blot analysis of GFP-tagged SSU processome components Utp5 (**B**), Rrp9 (**C**) and Rcl1 (**D**) in wild type or *npl3*∆ is shown. Hem15 or Grx4 served as loading controls. The cross-reaction bands are indicated with *. n = 3. (**E**) The amount of some SSU processome components is altered in the *NPL3* deletion strain. Protein levels in the western blots performed as in (B-D) were quantified and normalized to the loading control. *P < 0.05; **P < 0.01; ***P < 0.001. (**F**–**H**) Deletion of *NPL3* leads to synthetic growth defects in combination with mutants of SSU processome components. Ten-fold serial dilutions of the indicated strains were spotted onto agar plates that were incubated for 2 or 3 days at the indicated temperatures. Growth of *npl3*∆ is shown compared to the double mutant with *imp4—2* on full medium plates (**F**), with *nop1—3* on FOA plates that lead to a selection of cells that have lost the *URA3* containing plasmid (**G**) and upon downregulation of *UTP18* from a tet-off promoter on doxycycline containing plates (**H**). n = 3. (**I**) RNase-sensitive physical interaction of Npl3 with components of the SSU processome. Western blot analysis of co-IPs with GFP-tagged Rcl1, Utp18, Rrp9 and Utp5 are shown. A polyclonal anti-Npl3 antibody was used to detect co-precipitation of Npl3. Hem15 served as a negative control. n = 3 (**J**) Quantification of the western blots shown in H and replica experiments. *P < 0.05; **P < 0.01; ***P < 0.001. (K-M) Npl3 physically interacts with Nop1, Utp24 and Sas10 of the SSU processome. Western blots of co-IPs precipitating the GFP-tagged Nop1(K) and the GFP-tagged Npl3 (**L**, **M**) with Npl3 (**K**) or Myc-tagged Utp24 (**L**) or the Myc-tagged Sas10 (**M**) is shown. Hem15 served as a negative control. Vertical red lines indicate where the lysate and eluate samples required different exposure times during detection. In 4 K, the lysate lanes of Hem15 were exposed for 10 and the eluate lanes for 30 s. In 4L, the lysate lanes of GFP-Npl3 were exposed for 7 and the eluate lanes for 30 s

The SSU processome is an early pre-ribosomal intermediate assembled on the nascent pre-rRNA transcript from various sub-complexes (proteinaceous and snoRNP) and individual proteins. More than 50 proteins have been identified as SSU processome components. The findings that Npl3 associates with RNAPI, the rDNA and the 35S pre-rRNA [12] and (Fig. 1), suggest that it may associate with the SSU processome and/or influence its assembly. Western blot analysis of several protein components of the SSU processome (Fig. 4A) revealed that the UTP-A sub-complex protein Utp5 and the U3 snoRNP associated protein Rrp9 are present at lower levels in *npl3∆* compared to the wild type strain (Fig. 4B, C, E, Fig. S2E). By contrast, the mRNA abundance of *UTP5* and *RRP9* is only very slightly decreased in *npl3∆* (Fig. S2F, G). Therefore, we conclude that the reduced protein amount is likely due to protein degradation. Proteins are often destabilized if not incorporated into a complex, so impaired assembly of these proteins into the SSU processome in *npl3∆*, may reduce the stability of these SSU processome components. The instability of SSU processome proteins in cells lacking Npl3 does not seem to be a general phenomenon because the level of Rcl1, an RNA cyclase-like component of the SSU processome is unaffected in *npl3∆* cells (Fig. 4D, E).

To strengthen the notion of a function of Npl3 in SSU processome assembly, potential genetic interactions between *NPL3* and other SSU processome components were explored. *npl3∆* does not show profound growth defects compared to wild type. Mutant versions of the MPP10 complex component *IMP4* and the U3 snoRNP methyltransferase *NOP1* similarly showed only mild growth defects whereas a strain in which the UTP-B sub-complex protein *UTP18* was downregulated displayed strong growth defects. When *npl3*∆ was combined with any of the other mutations/depletions, the combination resulted in severe growth defects (Fig. 4F-H), suggesting a function of Npl3 related to the SSU processome.

To investigate potential physical interactions of Npl3 with proteins of the SSU processome, co-IP experiments were performed with various GFP-tagged SSU processome components and their interactions with Npl3 with and without RNase A treatment were analyzed. This revealed that Npl3 associates with Rcl1, Utp18, Rrp9 and Utp5 in an RNA dependent manner (Fig. 4I, J). Thus, either Npl3 requires the RNA binding to interact with these proteins or the interactions are indirect and mediated by the rRNA. Importantly, co-IPs of Npl3 with Nop1, the PIN domain endoribonuclease Utp24 and Sas10 showed RNA independent interactions (Fig. 4K-M), suggesting physical connections between Npl3 and these SSU processome proteins. Interestingly, the interaction between Npl3 and Nop1 is further increased when cells accumulate the 23S pre-rRNA upon rapamycin treatment (Fig. S2H, I), which might indicate that Npl3 is no bona-fide processosome component, but rather increasingly present on faulty rRNA. Together, these experiments indicate that Npl3 associates with the SSU processome and might contribute to its assembly.

**Fig. 5:**
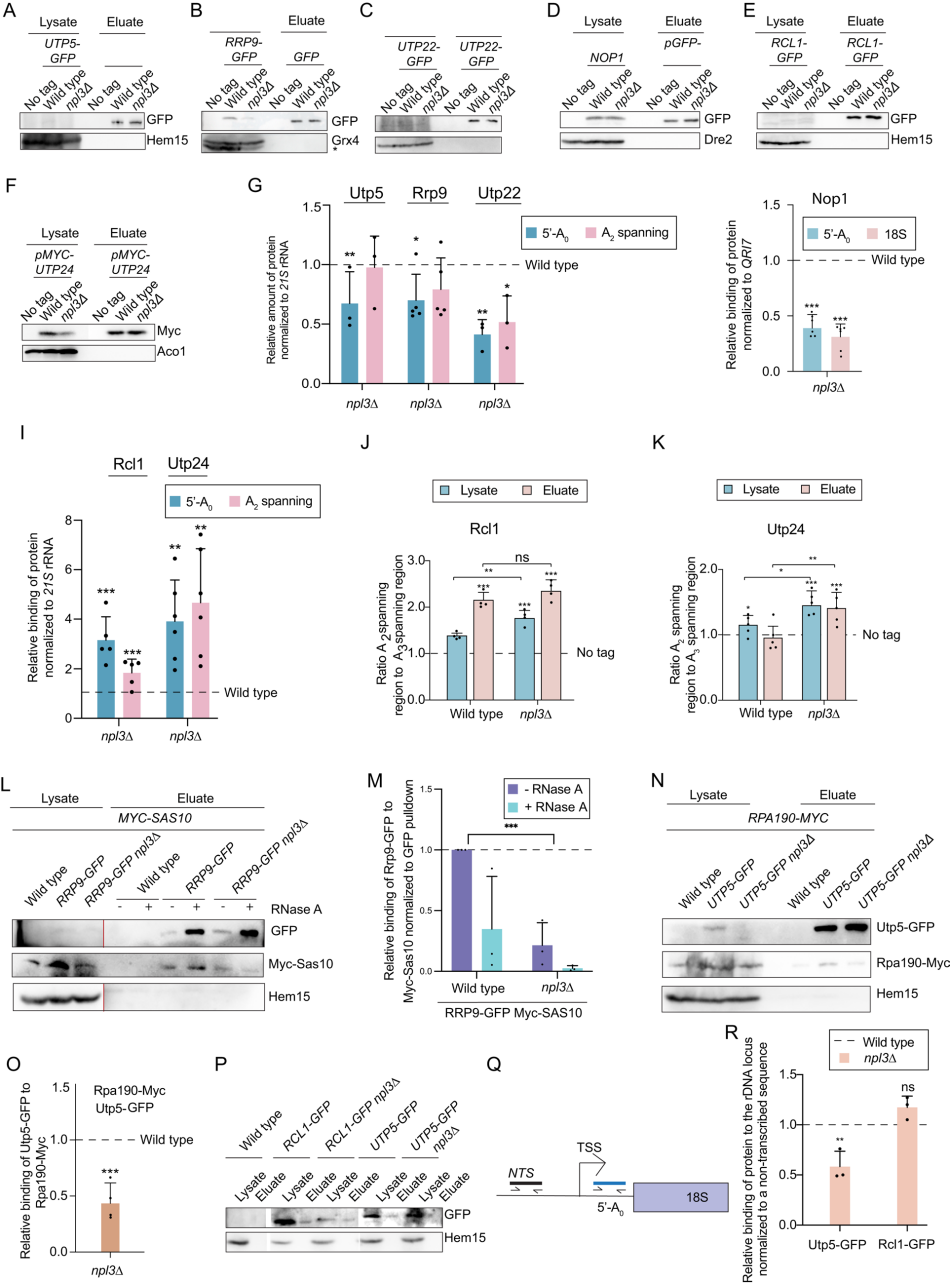
SSU processome formation on pre-rRNA is altered when *NPL3* is deleted**.** (**A**-**F**) RIP or CLIP experiments with GFP- or Myc-tagged SSU processome components were conducted. Western blot analysis shows an equal protein pulldown. A cross-reacting protein band is indicated with *. (**G**, **H**) qPCRs with primers targeting the ETS1, ITS1, or the 18S rRNA parts of the 35S rRNA reveal decreased binding of Utp5, Rrp9, Utp22 (**G**) and Nop1 (**H**) to the pre-rRNA in *npl3*∆. The binding was normalized to the mitochondrial 21S rRNA (**G**) or the mitochondrial *QR17* RNA (H). n = 5 Rrp9 RIP, n = 6 Utp5 RIP, n = 3 Utp22 CLIP and n = 6 Nop1 CLIP. (**I**) qPCRs with primers targeting the ETS1 and ITS1 of the 35S rRNA reveal increased binding of GFP-tagged Rcl1 and Myc-tagged Utp24 to the pre-rRNA in *npl3*∆. n = 4 Rcl1 RIP, n = 6 Utp24 RIP. *P < 0.05; **P < 0.01; ***P < 0.001. (**J**, **K**) The ratio of A_2_ to A_3_ spanning qPCRs revealed an increased binding of Rcl1 (**J**) and Utp24 (**K**) to the 23S rRNA. n = 4 Rcl1 RIP, n = 6 Utp24 RIP. (**L**) The interaction of Sas10 and Rrp9 is reduced in *npl3*∆. Western Blot analysis of a co-IP of Myc-Sas10 with Rrp9-GFP is shown in wild type and *npl3∆*. Hem15 served as a negative control. The lysates and eluates of Rrp9-GFP and Hem15 were detected using different exposure times indicated by the red vertical line (Rrp9-GFP: lysate 5 min, eluate 30 s; Myc-Sas10: lysate and eluate 5 min; Hem15: lysate 9 s, eluate 10 min). (**M**) SSU processome assembly is impaired when *NPL3* is deleted. Quantification of three independent experiments, one of which is shown in L. n = 3. *P < 0.05; **P < 0.01; ***P < 0.001. (**N**) The co-transcriptional recruitment of Utp5 is disturbed in *npl3*∆. A co-IP experiment was carried out using GFP-Selector beads to pull on GFP-tagged Utp5. Myc-tagged Rpa190 was detected using western blot analysis. Hem15 served as a negative control. (O) Binding of Utp5-GFP to Rpa190-Myc was calculated by relating the relative intensity of the Myc signal to the GFP-signal. n = 4 (P-R) ChIP experiments pulling on GFP-tagged Rcl1 or Utp5 in wild type or *npl3Δ* are shown. (**P**) Pulldown of GFP-tagged proteins was shown via western blot analysis. Hem15 served as a negative control. (**Q**, **R**) DNA isolation and subsequent qPCR analysis with primers binding to the ETS1 region or the non-transcribed sequence (*NTS*) (**Q**) upstream of the rDNA locus, was conducted. (**R**) The binding to the ETS1 in *npl3Δ* in three independent experiments, one of which is shown in P, was related to the binding in wild type and normalized to the *NTS*. n = 3

To further explore the notion that Npl3 contributes to SSU processome assembly, the associations of SSU processome components with the pre-rRNA were analyzed using RNA co-IP (RIP) experiments with or without UV crosslinking. Interestingly, while the binding of Utp5, Rrp9, Utp22 and Nop1 to the SSU pre-rRNAs was decreased in *npl3*∆ compared to wild type, the binding of Rcl1 and Utp24 was significantly increased (Fig. 5A-I). The qPCR results were normalized to the mitochondrial 21S rRNA or the mitochondrial mRNA *QRI7* in case of Nop1, which was found to artificially bind to 21S rRNA after lysis. The finding that some SSU processome components are reduced in their pre-rRNA binding when *NPL3* is deleted supports the requirement of Npl3 for proper SSU processome assembly. Interestingly, Utp24 and Rcl1, which accumulated on pre-rRNA in the absence of *NPL3* were suggested to interact with the 23S pre-rRNA [46, 50]. As this aberrant pre-rRNA accumulates when *NPL3* is deleted, the increased binding of these two proteins likely reflects their interaction with this transcript. To confirm the increased binding of Rcl1 and Utp24 to the 23S rRNA, the RNAs recovered with these two proteins were used for RT-qPCR experiments relating the A_2_ spanning and A_3_ spanning regions, which indicates the presence of the 23S pre-rRNA. As expected, accumulation of the 23S pre-rRNA in *npl3∆* compared to wild type was observed already in the lysate (Fig. 5J, K). Both Rcl1 and Utp24 strongly enriched the 23S pre-rRNA in the wild type and *npl3∆* strains, indicating that both proteins bind to the 23S rRNA independently of Npl3. As the MRP complex is responsible for the cleavage at A_3_ that generates the 23S pre-rRNA we carried out RIP experiments with MRP components in *npl3∆*. We found that Rmp1 and Snm1 show an increased binding to the A_3_ spanning region in *npl3∆* (Fig. S3A-D). This is similar to Rcl1 and Utp24 and it indicates that the 23S rRNA is still associated with Rcl1, Utp24 and the MRP complex in *npl3*∆, possibly awaiting disassembly prior to rRNA degradation.

Building on the finding that some SSU processome components are not optimally associated with the pre-rRNAs when Npl3 is lacking, we next explored whether the absence of Npl3 affects the interactions between different SSU processome components. Proper SSU assembly requires Sas10, which is important for protein rearrangements on the growing SSU processome and is therefore a central component [28]. To see whether the lack of *NPL3* affects Sas10 interactions within the SSU processome, co-IPs of the Rrp9 and Sas10 were carried out. This demonstrated that their interaction was significantly disturbed in the *npl3*∆ strain (Fig. 5L, M); only ~ 30% of the Sas10 recovered with Rrp9 in wild type cells was detected when Npl3 was missing. This shows the importance of Npl3 not only for loading of SSU processome proteins onto the pre-rRNA, but also for the assembly of proteins within the complex.

Most of the SSU processome proteins are recruited co-transcriptionally to the pre-rRNA. As Npl3 was suggested earlier to be involved in the co-transcriptional recruitment of proteins to RNAPII-synthesized mRNAs [51], it is possible that Npl3 might fulfil a similar role in the context of SSU processome assembly. As a component of the UTP-A sub-complex, one of the earliest proteins to be assembled onto the nascent pre-rRNA transcript is Utp5 (Fig. 4A). To determine if this protein is loaded properly by the RNAPI onto the pre-rRNA in *npl3∆*, co-IPs were performed to analyze the association of Utp5 and the largest component of RNAPI, Rpa190, in the presence and absence of Npl3. Strikingly, compared to wild type, the interaction of Utp5 with Rpa190 was reduced to approximately half in *npl3*∆ (Fig. 5N, O), indicating that defects in the recruitment of SSU processome components occurs already co-transcriptionally. It is possible that the role of Npl3 in co-transcriptional recruitment of SSU processome components to the nascent transcript is restricted to the very earliest recruited factors as ChIP-qPCR experiments demonstrated the reduced association of Utp5 with the rDNA locus in *npl3*∆, but revealed no change in the association of the later recruited SSU processome component (Fig. 5P-R, Source data 3).

Together, these results suggest that the knockout of *NPL3* perturbs recruitment of some SSU processome components onto the nascent pre-rRNA transcript. While this may reflect a direct role of Npl3 in the interaction of SSU processome proteins, alternatively, it could indicate the necessity of Npl3 association with the emerging 35S pre-rRNA during transcription to prevent possible misfolding of the nascent transcript and thereby, impaired loading of some SSU processome factors.

### Npl3 recruits the 3’ degradation machinery to the pre-rRNAs

In its function in mRNA surveillance, Npl3 participates in the quality control of 5’ capping; on correctly capped mRNAs, Npl3 recruits Mex67 for nuclear export and on transcripts with defective caps it retrieves the 5’exonuclease complex Rat1-Rai1 for degradation instead [6]. While Npl3 is required for nuclear export of pre-LSU, but not pre-SSU particles, it is possible that Npl3 plays a role in recruitment of the RNA decay machinery to aberrant pre-rRNAs to promote their nuclear degradation. Two main RNA degradation pathways exist in the nucleus; RNAs can be degraded either from the 5’ end by Rat1-Rai1 and/or from the 3’ end by the nuclear, Rrp6-containing exosome. Exosome-mediated RNA degradation requires co-factors such as the TRAMP complex [34], and a function of the TRAMP5–exosome complex in the degradation of the excised 5’-A_0_ fragment of the ETS1 and the 23S pre-rRNA has been described [26, 52]. Degradation of the 23S pre-rRNA from the 5’ end has so far not been observed, and consistent with the predominant 3’−5’ decay of this aberrant species, the 23S pre-rRNA did not accumulate in *rat1—1* mutant cells at the non-permissive temperature (Supplementary Fig. 4 A). By contrast, a slight, but not significant, accumulation of the 5’-A_0_ fragment of the ETS1 was observed (Fig. S4B, C), which is in line with a contribution of Rat1 to turnover of this excised spacer fragment. Notably, association of Rat1 with this fragment was not affected by the absence of Npl3 as shown in RIP experiments (Fig. S4D-F).

**Fig. 6:**
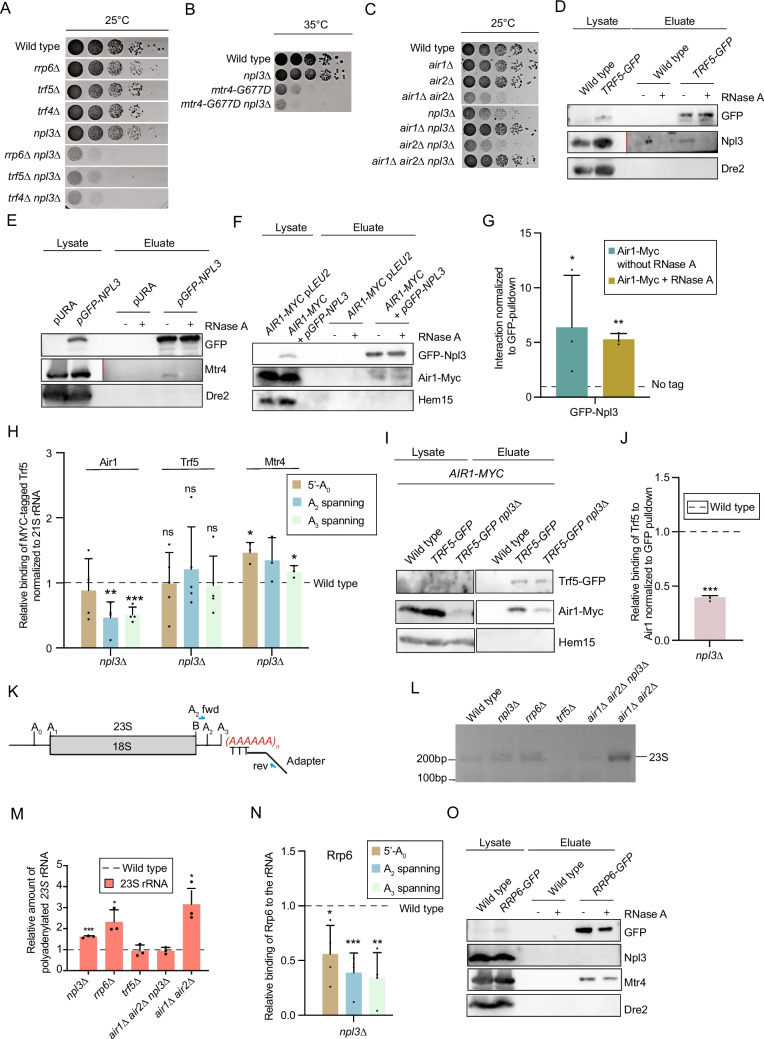
Npl3 is important for the recruitment of the 3’ −5’ degradation machinery to aberrant pre-rRNAs**.** (**A**-**C**) Genetic interactions of *NPL3* and genes encoding TRAMP/exosome complex components are shown. The indicated single, double or triple mutants were spotted in serial dilution onto (**A**-**C**) full medium agar plates incubated for three days. (**D**-**I**) Npl3 physically interacts with the TRAMP5-complex. Western blots of Npl3 co-IPs with and without RNase A treatment are shown for Trf5 (**D**) Mtr4 (**E**) and Air1 (**F**). Dre2 and Hem15 served as negative controls. The lysate and eluate of Npl3 from (**D**) and Mtr4 from (**E**) were detected using different exposure times, indicated by a red vertical line (Npl3: lysate 1 and eluate 15 s; Mtr4: lysate 11 and eluate 56 s). n = 3. (**G**) Quantification of the interaction between GFP-Npl3 and Air1-Myc related to wild type and normalized to the GFP pulldown is shown. n = 3. *P < 0.05; **P < 0.01; ***P < 0.001. (**H**) Air1 binding to pre-rRNA is significantly reduced when *NPL3* is deleted. RIP experiments with Air1 in wild type and *npl3*∆ and subsequent qPCRs with the indicated parts of the 35S rRNA were carried out. Relative binding to the RNA was normalized to the mitochondrial 21S rRNA. n = 3. *P < 0.05; **P < 0.01; ***P < 0.001. (**I**-**J**) Trf5 (**I**) and Mtr4 (**J**) binding to pre-rRNA is not altered or slightly elevated when Npl3 is missing. RIP experiments with Trf5-Myc (n = 6) and CLIP experiment of Mtr4-GFP (n = 4) were carried out. Subsequent qPCRs with indicated parts of the 35S rRNA followed. Binding was normalized to mitochondrial 21S rRNA. *P < 0.05; **P < 0.01; ***P < 0.001. (K-L) The interaction between Trf5 and Air1 is reduced in *npl3*∆. (**K**)Western blot analysis of Air1-Myc co- IPs with GFP-Selector beads pulling GFP-Trf5 are shown. Hem15 served as a negative control. n = 3. (**L**) Quantification of three independent experiments, one of which is shown in K. (**M**) Scheme for the conducted 3’ RACE experiment shown in M. An oligo(**T**) containing adapter primer was used with the A_2_ forward (fwd) primer to amplify polyadenylated 23S rRNA. (**N**) Increased amounts of polyadenylated 23S rRNA accumulate in *npl3*∆ and *air1∆ air2∆*. 3’ RACE was caried out with RNA isolated from the indicated strains. RNA isolations were reverse transcribed with an oligo(**T**) containing adapter primer as depicted in M. Subsequent PCRs were conducted using primers that bind to the adapter sequence and an internal sequence (A_2_ fwd) that anneals upstream of the A_2_ cleavage site. The 3’ RACE PCR products are shown on an agarose gel. Deletion of *RRP6* served as a positive control for accumulation of polyadenylated 23S rRNA and deletion of *TRF5* as a negative control. n = 3. (**O**) Quantification of three independent experiments, one of which is shown in N. (**P**) Nuclear exosome recruitment to rRNA is disturbed in *npl3*∆. CLIP experiment with Rrp6 is shown in the indicated strains for products with primers binding the ETS1 or spanning the A_2_ cleavage site. n = 4. *P < 0.05; **P < 0.01; ***P < 0.001. (Q) Npl3 does not interact with the exosome component Rrp6. Western-blots of co-immunoprecipitated Npl3 and the positive control Mtr4 with Rrp6-GFP are shown. n = 3

We therefore focused on investigating a potential contribution of Npl3 to the 3’−5’ degradation of the 23S pre-rRNA. First, potential genetic and physical interactions between Npl3 and TRAMP or exosome complex components were examined. While strains lacking *NPL3*, *RRP6*, *TRF4* or *TRF5* grew as wild type at 25 °C, upon crossing of relevant strains, strong growth defects were observed for *npl3∆* with *rrp6*∆, *trf4*∆ and *trf5*∆ (Fig. 6A, B, Fig. S5A). While a temperature-sensitive mutant of *MTR4* already lead to a strong growth defect at the restrictive temperature, this was further exacerbated by *npl3∆*. These results indicate a genetic relationship between Npl3 and the 3’−5’ degradation machinery. Also, the deletion of *AIR2* led to a slight growth defect in combination with *npl3∆,* which is stronger than the single mutants alone*.* By contrast deletion of *AIR1* together with *npl3*∆ did not affect growth, possibly because Air2 is able to compensate the loss of Air1 in *npl3∆*. Strikingly, the triple knock-out grew better than the single deletions alone on YPD-plates and the double knock out *air1∆ air2∆,* which shows significant growth defect (Fig. 6C), indicating that their combined loss does not restrict cells in their growth and that the requirement for both Air1 and Air2 entirely depends on the presence of Npl3. We hypothesize that the triple mutant strain might rather use a completely different pathway for recruiting the degradation machinery, independently of Npl3 and both Air proteins, which is why such complete shift might be tolerable.

To detect potential physical interactions between Npl3 and the TRAMP5 complex components Trf5, Mtr4 and Air1, as well as the additional TRAMP4 complex components, Trf4 and Air2, co-IP experiments were performed in the presence and absence of RNase. While all proteins showed RNase-sensitive interactions with Npl3 (Fig. 6D-E; Fig. S5B, C), an RNA-independent interaction was only detected between Npl3 and Air1 of the pre-rRNA degrading TRAMP5 complex (Fig. 6F, G). These data reveal a contact between Npl3 and Air1 that is mediated by protein–protein interactions and indicate that Npl3 either must be RNA bound to contact the other proteins of the TRAMP complexes or that they are located at the same time on one RNA substrate.

To analyze whether Npl3 can recruit the 3’−5’ degradation machinery to the aberrant 23S pre-rRNA and the 5’-A_0_ fragment of the *5’ETS* that awaits decay, RIP-qPCR experiments were performed. Interestingly, the association of Air1 with the A_2_ spanning region, either of the 35S pre-rRNA or of the 23S pre-rRNA that accumulates in *npl3∆* (Fig. 2B, 3B), was reduced to half, which suggests a reduced association of Air1 with the faulty 23S pre-rRNA when Npl3 is missing (Fig. 6H; Fig. S5D). Notably, while association of Air1 with the 23S pre-rRNA was significantly reduced, the association of Trf5 and Mtr4 was unaffected or even significantly enriched (Fig. 6H, Supplementary Fig. 5 E–G). Co-IP experiments with Air1 and Trf5 further showed that the deletion of *NPL3* impairs their interaction, with less than half the amount of Air1 recovered with Trf5 in *npl3*∆ compared to wild type (Fig. 6I, J, Source data 4). However, the result of this experiment must be seen with caution, because less Air1 is present in *npl3∆*, which might indicate an instability of the protein, when Npl3 is absent and Air1 not in a complex. Regardless of the reason, why Air1 is reduced in *npl3∆*, the absence of Npl3 affects the formation of the TRAMP5 complex. For the TRAMP4 complex components Air2 and Trf4, the same trend in binding to the A_2_ spanning region of the pre-rRNA was detectable as for Air1 and Trf5, respectively (Supplementary Fig. 5 H–K), which might be due to a potential back up function. These results suggest that upon *NPL3* deletion, assembly of the TRAMP complex is affected as well as its association with pre-rRNAs.

Association of the poly(A) polymerases Trf5 and Trf4 with pre-rRNAs is not affected by deletion of *NPL3*, but as interaction of the Air1/2 proteins with the pre-rRNA is perturbed, we investigated whether polyadenylation of the 23S rRNA was affected in *npl3*∆. 3’RACE experiments were conducted by initiating cDNA synthesis with an oligo(T) containing a PCR adapter sequence and subsequently performing PCR-based amplification of the 23S rRNA end using a forward primer binding upstream of the A_2_ cleavage site and a reverse primer binding the adapter sequence (Fig. 6K). As expected, a polyadenylated product was very weakly detected in wild type cells; in this strain A_2_ cleavage is only rarely bypassed for cleavage at A_3_ so very few 23S pre-rRNAs are produced, and those that are, are efficiently degraded. However, polyadenylated 23S pre-rRNA was clearly detectable in *rrp6*∆ when the 23S pre-rRNA cannot be easily degraded (Fig. 6L, M). No polyadenylated 23S pre-rRNA was detected in *trf5*∆ as the enzyme responsible for pre-rRNA polyadenylation was lacking. Most importantly, polyadenylated 23S pre-rRNA was well detectable in *npl3*∆, showing that the 23S pre-rRNA that accumulates in *npl3*∆ is polyadenylated, despite the Air proteins not being recruited properly. It has been reported that the Air proteins are involved in the recruitment of the Trf proteins to transcripts prior to Mtr4 joining and exosome-mediated degradation [34] [35]. Interestingly, as polyadenylated 23S pre-rRNA was enriched in the *air1∆ air2∆* strain, our data indicate that this aberrant pre-rRNA can be polyadenylated in the absence of the Air-proteins, but that degradation seems to be defective (Fig. 6L). This indicates that the Air proteins are not required for the RNA-association or enzymatic activity of Trf5 and rather suggests that they are responsible for recruitment of the degradation machinery. Therefore, we investigated recruitment of the exosome in *npl3*∆, in which the Air proteins display reduced pre-rRNA association, and indeed observed decreased Rrp6 interaction with the 23S rRNA (Fig. 6N, Fig. S5L). These data suggest a role for Npl3 in recruitment of the Air proteins, and in turn, the exosome to degrade the aberrant pre-rRNA. As no physical association between Npl3 and Rrp6 was detected by co-IP (Fig. 6O), it is likely that the nuclear exosome associates with the 23S pre-rRNA after Npl3 has dissociated. It remained puzzling, though that the growth rate of the triple mutant *air1∆ air2∆ npl3∆* resembled that of wild type (Fig. 6C). Therefore, we investigated the oligoadenylation of the 23S rRNA in this strain. Remarkably, we found that in *air1∆ air2∆ npl3∆*, oligoadenylation of the 23S rRNA went back to wild type levels (Fig. 6L, M). This suggests that alternative pathways for 23S rRNA elimination must exist. This would also explain the wild typic growth rate of the triple mutant (Fig. 6C).

**Fig. 7:**
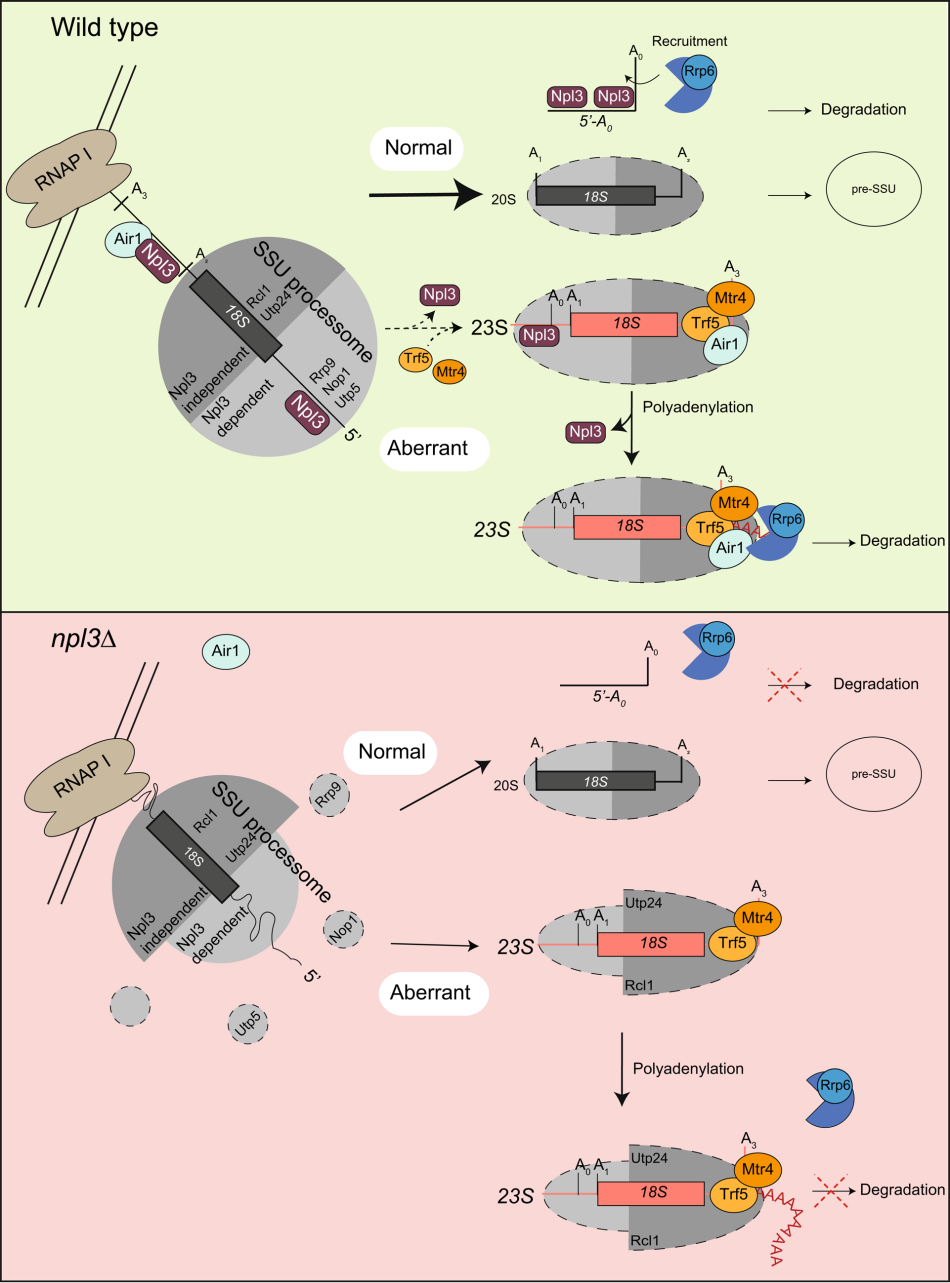
Model for the Npl3 surveillance function in pre-rRNA quality control. Top**:** Npl3 is recruited co-transcriptionally to the pre-rRNA enabling efficient recruitment of several SSU processome components, including Utp5, Nop1 and Rrp9. The stepwise assembly of the SSU processome is monitored by the guard protein Npl3, while bound to Air1 of the TRAMP complex. To correctly process ETS1 fragments, Npl3 supports the recruitment of the nuclear Rrp6-containing exosome for degradation. On aberrant 23S pre-rRNAs, Npl3 promotes formation of the TRAMP5 complex and subsequent recruitment of the exosome for degradation. **Bottom:** In the absence of Npl3, the ETS1 fragments are not efficiently degraded and several SSU processome proteins, such as Utp5, Rrp9 and Nop1 are not recruited properly to the pre-rRNA. Their failure to integrate into the SSU processome leads to degradation of these proteins. In *npl3*∆, increased cleavage at A_3_ occurs due to perturbed SSU processome assembly and the resulting 23S pre-rRNA accumulates. Although polyadenylated by Trf5, the 23S pre-rRNA is not degraded properly, because the Npl3-mediated loading of Air1 to the rRNA is reduced, which leads to an impaired recruitment of the exosome

Taken together, our data suggest that Npl3 is recruited co-transcriptionally to the emerging pre-rRNA (Fig. 7). In the presence of Npl3, the SSU processome assembles correctly, possibly due to minimization of incorrect pre-rRNA folding allowing cleavage of the 35S pre-rRNA occur stepwise in the correct order from A_0_ to A_2_. Degradation of the resulting 5’-A_0_ 5’ ETS fragment is promoted by Npl3, likely by recruitment of the 3’−5’ nuclear exosome. Correct assembly of the SSU processome allows subsequent SSU maturation steps and the pre-SSU is exported into the cytoplasm. When SSU processome assembly and pre-rRNA processing do not occur in a timely manner, the 23S pre-rRNA is generated. This dead-end product is polyadenylated by the TRAMP5 complex and degraded by the exosome. In the presence of Npl3, this degradation machinery assembles correctly and ensures efficient elimination of the 23S pre-rRNA. However, in the absence of Npl3, assembly of the SSU processome is perturbed and pre-rRNA processing is impaired, leading to increased production of the 23S pre-rRNA. Although adenylated by Trf5, the accumulated 23S pre-rRNA is not degraded efficiently because recruitment of Air1 is disturbed. The decreased association of Air1 with the polyadenylated 23S pre-rRNA results inhibits recruitment of the Rrp6-containing exosome and the 23S rRNA persists. Importantly, our data furthermore show that the guarding function of Npl3 is not limited to mRNA maturation but is also utilized in the context of pre-rRNA surveillance. Via its ability to recruit RNA degrading enzymes and monitor proper ribonucleoprotein complex assembly, this guard protein helps to ensure the integrity of various RNPs in cells.

## Discussion

Nascent RNAs are typically bound by multiple RNA-binding proteins acting as chaperones for RNA folding and coordinating RNA processing and RNP maturation steps. Pre-mRNA maturation is surveilled by a family of RNA-binding guard proteins, which includes Npl3. Guard proteins are loaded co-transcriptionally onto pre-mature transcripts by the CTD of RNAP II and retain them in the nucleus until processing is completed [5, 18, 45]. Guard protein-mediated surveillance ensures that Mex67 binds only after processing events are completed, guaranteeing that exclusively mature mRNAs leave the nucleus for translation in the cytoplasm.

Defects in pre-rRNA processing have been observed in cells lacking Npl3 [36], but to date, it has remained unclear whether these arise as an indirect consequence of perturbed mRNA biogenesis or reflect multifunctionality of Npl3, with the guard protein also fulfilling a direct role in pre-rRNA surveillance. Our study provides several evidence that Npl3 not only surveilles pre-mRNA processing but also rRNA biogenesis. Firstly, Npl3 not only associates with pre-mRNAs, but also directly binds to pre-rRNAs [9, 12]. Additionally, we demonstrate that, analogous to pre-mRNAs, this association takes place co-transcriptionally (Fig. 1). Npl3 physically interacts with RNAP I (Fig. 1E-G) and binds to the transcribed region of the rDNA that encodes the 5’ region of the pre-rRNA. Further supporting the co-transcriptional association of Npl3 with early pre-ribosomal particles, physical interactions of Npl3 with proteins of the early SSU processome sub-complexes (e.g. Utp5 of the UTP-A sub-complex) were observed (Fig. 4I-M). Interestingly, these interactions were overall rather slight and not comparable to the interactions detected between processosome components, although RNAPI transcription events are by far the most occurring. This is intriguing and fits well to the suggested role of Npl3 in rRNA quality control, where rather transient interactions would be expected. The physical association of Npl3 with RNAP I, rDNA, pre-rRNAs and early ribosome assembly factors strongly supports a direct role in ribosome biogenesis and/or quality control. Interestingly, Npl3 also interacts with SSU processome components recruited at later stages, such as Utp24, Sas10 and Rcl1, suggesting that Npl3 has prolonged association with the pre-rRNAs. As Npl3 is highly expressed with around 30,000 molecules per cell (https://www.yeastgenome.org), it is possible that several molecules of Npl3 may be associated with each nascent pre-ribosomal particle. Its presence in ribosomal profiles is mostly detectable on pre-40S particles, supporting a role during maturation of the small ribosomal subunit (Fig. 1K). Due to its multiple other functions in mRNA biogenesis, much of the protein is also present as a non-pre-ribosome-associated population. Drawing parallels to the model proposed for Npl3-mediated pre-mRNA surveillance, it is conceivable that binding of Npl3 to several positions across the emerging pre-rRNA transcript may limit the formation of inappropriate secondary structures, thus facilitating timely recruitment of SSU processome components. Indeed, compared to wild type, in *npl3*∆, the binding of several early SSU processome components (e.g. Utp5, Rrp9, Utp22 and Nop1) to the pre-rRNA was reduced (Fig. 5G, H), demonstrating perturbed SSU processome assembly in the absence of the guard protein. The binding of multiple Npl3 molecules to the pre-rRNA would be in line with the RNase-sensitive nature of many of the interactions of Npl3 with SSU processome components (Fig. 4I, J).

Defects in SSU processome assembly typically impair pre-rRNA processing at sites A_0_, A_1_ and A_2_, leading to cleavage at site A_3_ by RNase MRP and accumulation of the aberrant 23S pre-rRNA [26]. The level of the dead-end 23S pre-rRNA, which is normally eliminated from cells, strongly increases in cells lacking Npl3 (Fig. 2A-E). This accumulation likely reflects both the impaired SSU processome assembly in *npl3*∆ as well as the contribution of Npl3 to recruitment of the RNA degradation machinery to this aberrant pre-rRNA species. Utp5, Rrp9, Utp22 and Nop1 are all required for the cleavages at sites A_0_ to A_2_ (Fig. 5G, H) so their decreased association with pre-rRNAs and with each other in cells lacking Npl3 rationalizes the accumulation of the 23S pre-RNA in this strain. In addition, the increased association of the MRP complex with the pre-rRNA is detected in *npl3*∆, is in line with enhanced cleavage at site A_3_ to generate the 3’ end of the 23S pre-rRNA (Fig. S3A-D). Interestingly, the SSU processome components that associate with the pre-rRNA independently of Npl3 remain associated with the aberrant 23S pre-rRNA in the *NPL3* deletion strain (Fig. 5I-K). The binding of Npl3 proteins to the emerging pre-rRNA therefore appears to be important for the stepwise recruitment/binding of SSU processome components, and it is likely this guard protein also dissociates in a stepwise manner upon correct association/positioning of the processing factors and the ribosomal proteins. It is likely through this role in orchestration of SSU processome assembly that Npl3 influences pre-rRNA processing events. Only if the stepwise assembly is disturbed, Npl3 persists on the pre-rRNA and promotes degradation of the formed 23S pre-RNA. Its increased presence on the 23S pre-rRNA upon rapamycin treatment supports this role (Fig. 2H). In contrast to its stepwise removal from the maturing 18S rRNA precursors, Npl3 remains bound to the cleaved ETS1 fragment, where it promotes recruitment of the degradation machinery and to eliminate this excised spacer fragment (Fig. 2I-O, 6P). On the correctly maturing 18S rRNA precursors, Npl3 dissociates to avoid recruitment of the degradation machinery. This fits well with an earlier study, in which it was revealed that Npl3 does not accompany the pre-SSU into the cytoplasm for final maturation [12]. The retention of Npl3 on aberrant pre-rRNAs when maturation steps were not completed properly, promotes recruitment of the RNA degradation machinery to eliminate the faulty particles prior to their nuclear export.

Whether Npl3 is actively involved in the recruitment of SSU processome components remains unclear. While it is possible that Npl3 directly influences the recruitment of SSU processome components, the finding that many interactions between Npl3 and SSU processome components are RNase-sensitive makes this unlikely. Binding of Npl3 might rather chaperone pre-rRNA folding to facilitate association of the SSU processome proteins. Upon correct positioning of SSU processome proteins, Npl3 might dissociate in a “switch-like” mechanism reminiscent of the switch-like mechanism observed during pre-mRNA quality control. In the context of pre-mRNAs, Npl3 binding can lead to recruitment of the degradation machinery, which is only prevented when this guard protein is covered by Mex67. Binding of Mex67 represents the switch from degradation to export of the mRNA to the cytoplasm. On pre-rRNAs, the switch seems to be the dissociation of Npl3 and Air1 from correctly processed pre-rRNAs to allow their ongoing maturation versus the persistent binding of Npl3 and Air1 to aberrant pre-ribosomes containing the 23S pre-rRNA leading to TRAMP-complex formation and RNA decay. We therefore propose that Npl3 surveys the early steps of pre-ribosome maturation until it is assured that they are successfully accomplished, whereupon Npl3 fully dissociates from the SSU processome.

To aberrant pre-mRNAs, Npl3 recruits the 5’ degradation factors Rat1-Rai1 [6], but this does not seem to be the case for faulty pre-rRNAs (Fig. S4). Rather, Npl3 is involved in the 3’ to 5’ directed degradation of pre-rRNAs via the TRAMP5 complex and the nuclear exosome (Fig. 6). Such 3’−5’ orientated degradation of the 23S pre-rRNA was previously suggested [26]; it is possible that Rat1 is not present in the nucleolus in sufficient amounts to counter the 23S pre-rRNA production, but alternatively, the presence of SSU processome components bound to the 5’ region of the pre-rRNA may also prevent degradation of the 23S pre-rRNA in the 5’−3’ direction. Either way, this demonstrates that Npl3 is not only able to recruit the 5’degradation machinery, as in the case of pre-mRNAs [6] but also the 3’ degradation complexes for the turn-over of aberrant pre-rRNAs (Fig. 6). A similar flexibility was also shown for the guard proteins Gbp2 and Hrb1, which recruit the TRAMP and exosome complexes for the degradation of unspliced pre-mRNAs in their nuclear quality control function and other degradation factors in their cytoplasmic function in the nonsense mediated decay (NMD) [40]. In the context of NMD, Gbp2 recruits the cytoplasmic 3’ degradation machinery composed of the Ski-proteins and the exosome. During NMD, Hrb1 recruits the 5’ degradation factors Dcp1 and Dcp2, that prepare the RNA for the 5’ exonuclease Xrn1 [13]. Thus, it seems that guard proteins can interact with multiple degradation factors and can support both the 5’−3’ and the 3’ −5’ degradation of different RNAs.

In the nucleolus, the TRAMP5 complex is predominant over TRAMP4 for 23S pre-rRNA degradation [35]. Interestingly, our data show that Npl3 can interact with components of both the TRAMP4 complex (Air2 and Trf4) and components of the TRAMP5 complex (Air1 and Trf5; Fig. 6D, F and Fig. S5B, C). This is in agreement with mass spectrometry analysis of Trf5 and Tr4 pulldown experiments published recently [35]. The association of Npl3 with the TRAMP4 complex could suggest that this guard protein has another function in the 3’−5’ degradation of pre-mRNAs in addition to its function in pre-rRNA surveillance. Indeed, Npl3 was suggested to bind at the 3’ end of mRNAs where it was reported to act antagonistically in 3’ end formation [10, 53, 54]. Therefore, it is well conceivable that it has a quality control function also at the 3’ end of mRNAs in which it could recruit the TRAMP4 and exosome complex.

The function of Npl3 in the degradation of faulty pre-rRNA appears to be to tether Air1 to the pre-rRNA (Fig. 6G, H). The detection of a physical interaction between Npl3 and Air1 that is RNA independent could suggest that these proteins interact before they associate RNA. It is further possible that this interaction might by influenced by the post-translation modification status of Npl3 as binding of Air1/2 to the methyltransferase Hmt1 was reported to inhibit arginine methylation of Npl3 [55]. Whether all bound Npl3 proteins are in a complex with Air1 remains unclear as Air1 is expressed at a lower level than Npl3. Previous models propose that the Air proteins bind RNAs and are required for the subsequent recruitment of the Trf proteins and thereafter Mtr4 before the exosome joins the complex for degradation [34, 35]. However, our results indicate Air1-independent binding of Trf5 to the 23S pre-rRNA. Although Air1 binding was reduced in *npl3*∆, this was not the case for Trf5 (Fig. 6H), indicating that Trf5 can still associate with the 23S pre-rRNA, which is present at an elevated level in *npl3*∆. Furthermore, the absence of Air1 and Air2 did not alter the polyadenylation of the 23S pre-rRNA (Fig. 6L, M), suggesting that the Air proteins are not a pre-requisite for polyadenylation by the Trf proteins. Furthermore, we show that the polyadenylation of the 23S rRNA is not disturbed but rather enhanced in *air1∆ air2∆* cells (Fig. 6M), suggesting that the Air proteins are possibly not the only Trf co-factors. Our data suggest that the function of the Air proteins in the TRAMP-complex mediated 3’ degradation of the 23S pre-rRNA is the recruitment of the exosome, because Rrp6 is less associated with this pre-rRNA in *npl3*∆, where Air1 binding is perturbed (Fig. 6N). As Npl3 shows no interaction with Rrp6 (Fig. 6O), we conclude that the Air1 protein, which is reduced in its pre-rRNA-binding when *NPL3* is deleted, is required for capturing the exosome.

Based on our data, we propose the following model for guard protein-mediated pre-rRNA quality control (Fig. 7). Npl3, possibly in multiple copies and potentially associated with Air1, is co-transcriptionally loaded onto the nascent 35S pre-rRNA. Through its RNA binding capability, Npl3 may prevent premature pre-rRNA folding, allowing the timely recruitment and positioning of SSU processome proteins. In case SSU processome assembly and pre-rRNA maturation occur correctly, Npl3 is released and with it, possibly also the bound Air1. However, in case the SSU processome formation and pre-rRNA encounter difficulties, Npl3-independent cleavage of the pre-rRNA at the A_3_ site results in production of the dead-end 23S pre-rRNA. The presence of the Npl3-Air1 complex on this aberrant pre-rRNA promotes TRAMP5 complex formation, leading to dissociation of Npl3 and recruitment of the nuclear exosome. The 23S pre-rRNA is polyadenylated by Trf5 independently of the Air proteins and TRAMP5 complex formation, although these factors are necessary for degradation of the faulty pre-rRNA. As neither Npl3 nor the Air proteins are essential and the poly(A)-marked 23S pre-rRNA can be degraded in their absence, although to a lesser extent, it seems likely that other factors exist that can also recruit the degradation machinery. Whether these are other guard proteins, such as Gbp2 and Hrb1, or other factors remains to be determined.

Together, these data reveal a novel function of Npl3 in pre-ribosome surveillance, demonstrating that this guard protein is multifunctional and not restricted to mRNA quality control. Future research will determine whether also other RNA species undergo surveillance by Npl3 and the other guard proteins.

## Supplementary Information

Below is the link to the electronic supplementary material.

Supplementary file1 (PDF 5885 KB)

## Data Availability

The datasets generated and/or analyzed for this study are stored at the “Gesellschaft für wissenschaftliche Datenverarbeitung mbH Göttingen” (GWDG). The RNA-seq data are available under accession number GSE268140 in the GEO database.
