# Supplementary material for: Ribosomal RNA processing undergoes surveillance by the mRNA guard protein Npl3
Source: Cell Mol Life Sci. 2026 May 19;83(1):209. doi: 10.1007/s00018-026-06246-6 (PMC13187095; doi:10.1007/s00018-026-06246-6)
Supplement: Supplementary file 1 — Supplementary file1 (PDF 5885 KB) [file 18_2026_6246_MOESM1_ESM.pdf]

Ribosomal RNA processing undergoes surveillance by the mRNA guard protein Npl3

- SUPPLEMENTARY INFORMATION -

Anne-Sophie Lindemann[1], Fei Yu[1], Yawen Duan[1], Ivo Coban[1], Ulla-Maria Schneider[1], Jan-Philipp Lamping[1], Ali Khreiss[2], Katherine E. Bohnsack[2] and Heike Krebber[1*]

[1]Abteilung für Molekulare Genetik, Institut für Mikrobiologie und Genetik, Göttinger Zentrum für Molekulare Biowissenschaften (GZMB), Georg-August Universität Göttingen, Göttingen, Germany
[2]Institut für Molekularbiologie, Universitätsmedizin Göttingen, Göttingen, Germany

[*]Correspondence: heike.krebber@biologie.uni-goettingen.de

Keywords: rRNA maturation / rRNA processing / RNA quality control / Ribosome biogenesis / RNA degradation /rDNA / RNA polymerase I /TRAMP complex /RNA exosome / 23S rRNA / ETS1

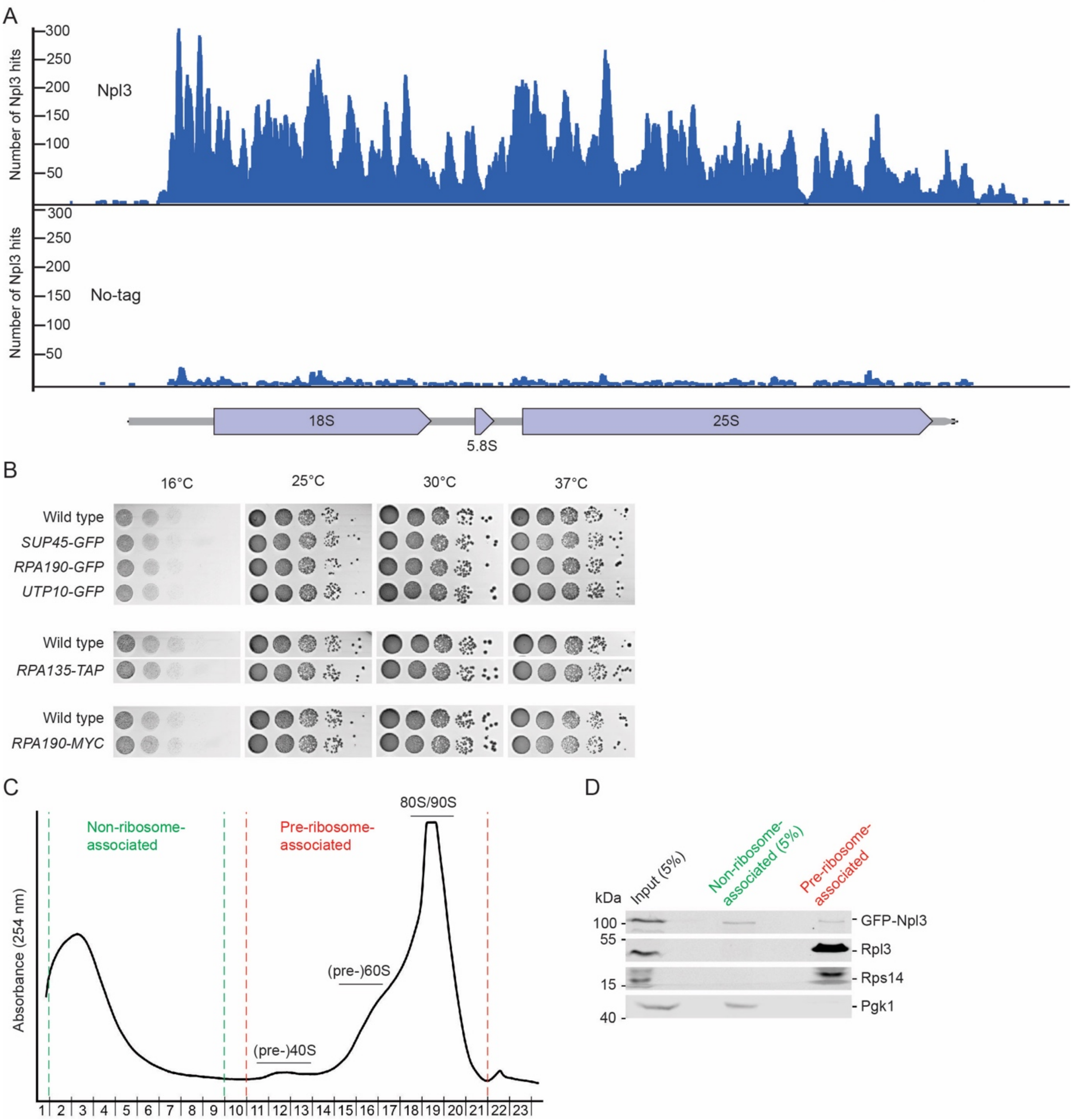

Supplementary Figure 1: (Related to Figure 1) (A) Npl3 CRAC data shows binding over the whole 35S rRNA transcript. Npl3 CRAC data (8), (Npl3:GSM1943527, No-tag: GSM1943525) was analyzed for the 35S rRNA. Npl3 occupancy is visualized over the 35S rRNA with the software IGB. Top: Npl3 Bottom: No-tag control, n=1 (B) GFP-tagged strains are functional. Growth analysis of the GFP-tagged strains is shown in comparison to wild type. The indicated endogenously tagged strains were spotted in 10-fold serial dilution onto full medium agar plates and incubated for 3 days at the indicated temperatures. (C, D) A portion of Npl3 co-migrates with pre-ribosomal particles in sucrose density gradients. (C) The absorbance profile reflects RNA content. (D) The fractions containing non-ribosome-associated Npl3 (Fractions 2-9) were pooled as well as the fractions containing pre-ribosome-associated Npl3 (Fractions 11-21), and the proteins in these two pools were analyzed via western blot (5% input, 5% non-ribosome-associated and 100% pre-ribosome associated). Western blots show the amounts of the indicated proteins in fractions containing the non-ribosomal and pre-ribosomal complexes. As similar band intensities are observed for GFP-Npl3 in the non-ribosome-associated pool and the pre-ribosome-associated pool, this indicates that approximately 5% of GFP-Npl3 co-migrates with pre-ribosomes in sucrose density gradients. n=2.

Supplementary Figure 2

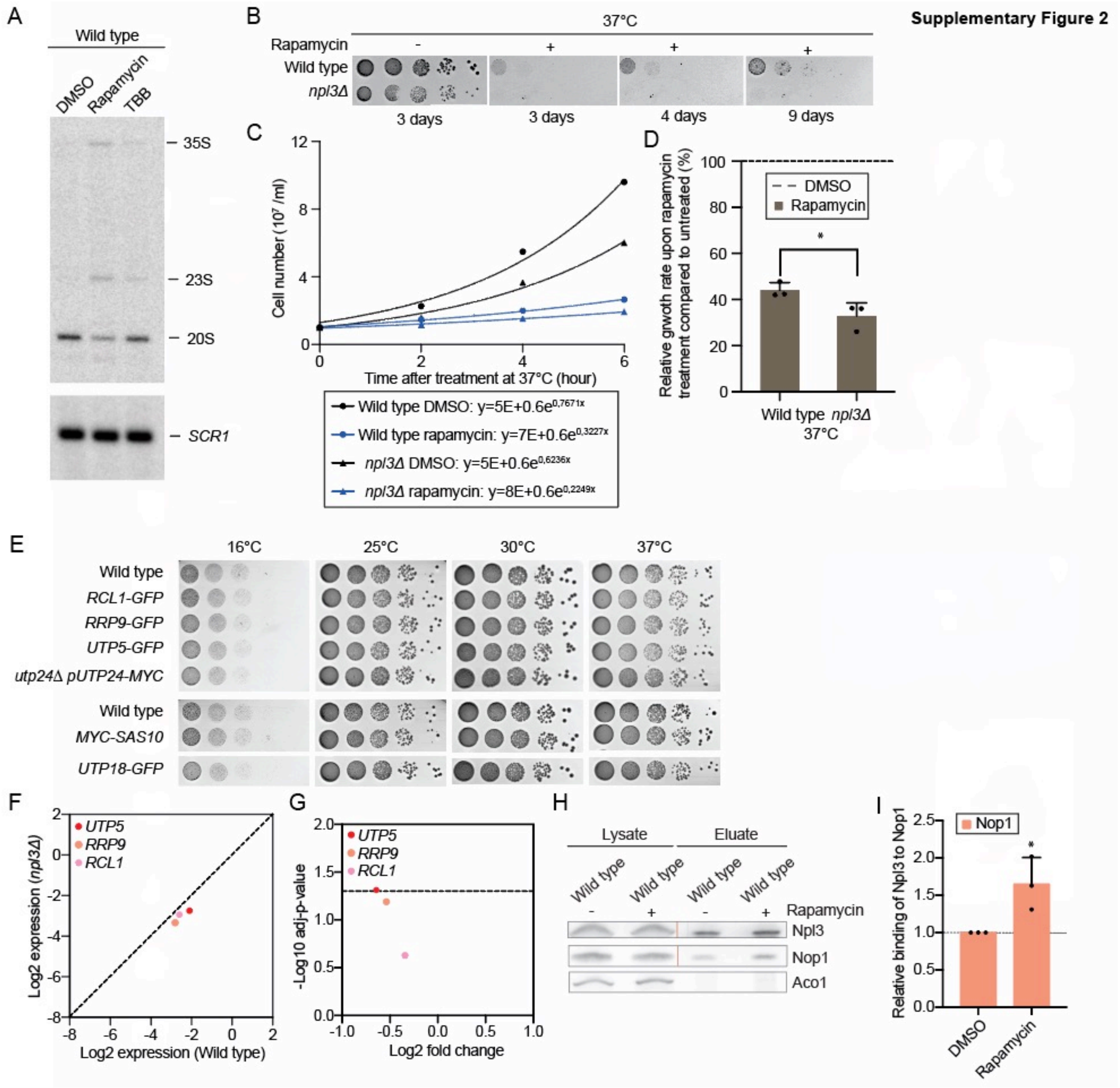

Supplementary Figure 2: (Related to Figure 2 and 4) (A) Northern blot analysis of RNA extracted from untreated and rapamycin treated wild-type cells using a probe hybridizing to the 5' end of ITS1 shows accumulation of the 23S pre-rRNA in response to rapamycin. *SCR1* served as a loading control. n=3. (B-D) Deletion of *NPL3* leads to a growth defect at 37°C upon rapamycin treatment. (B) Ten-fold serial dilutions of the indicated strains were spotted onto YPD plates containing either DMSO or rapamycin (200 ng/ml) that were incubated at 37°C for the indicated days. n=3. (C, D) Growth analysis of wild type and *npl3*Δ cells was conducted in liquid YPD medium containing either DMSO or rapamycin (200 ng/ml). (C) Growth curves of wild type and *npl3*Δ cells. Cells were grown in liquid YPD medium to log-phase and diluted to 1 x 10^7 cells /ml. Either DMSO or rapamycin were added and cells were incubated at 37°C. The cell number was determined every two hours and an exponential trendline was generated with an equation to represent the growth rate. (D) The relative growth rate derived from (C) is shown. n=3. (E) GFP-tagged strains are functional. Growth analysis of the GFP- or Myc-tagged strains is shown in comparison to wild type. The indicated endogenously tagged strains were spotted in 10-fold serial dilution onto full medium agar plates and incubated for 3 days at the indicated temperatures. (F, G) The mRNA levels of SSU processome components in *npl3*Δ are similar to those seen in wild type. Tilling microarray data from (9) were analyzed for the three mRNAs *UTP5*, *RRP9* and *RCL1*. (F) The scatter plot shows the total signal for each mRNA. Intensities are plotted on a log2 scale and the dashed line indicates equal intensity in the wild type and *npl3*Δ strains. (G) The volcano plot shows differentially expressed mRNAs in *npl3*Δ*.* The x-axis shows the log2 fold change, which indicates a reduced expression (negative) or an increased expression (positive). The y-axis is the negative log10 p-value, adjusted to false discovery rate (FDR). The dashed line is the threshold of a significant differential expression in *npl3*Δ ($p<0.05$) and mRNAs above the line are significantly decreased. (H, I) The Npl3 binding to Nop1 of the SSU processome increases upon rapamycin treatment. Cells were grown to log-phase and treated with either DMSO or rapamycin

(200 ng/ml) for 1 h. (H) Western blots of co-IPs precipitating Npl3 is shown. Aco1 served as a negative control. Vertical black lines indicate where the lysate and eluate samples required different exposure times during detection. The lysate lanes of Nop1 were exposed for 10.8 and the eluate lanes for 30 seconds. (I) Quantification of the western blot shown in J and replica experiments. n=3. *P < 0.05; **P < 0.01; ***P < 0.001.

Supplementary Figure 3

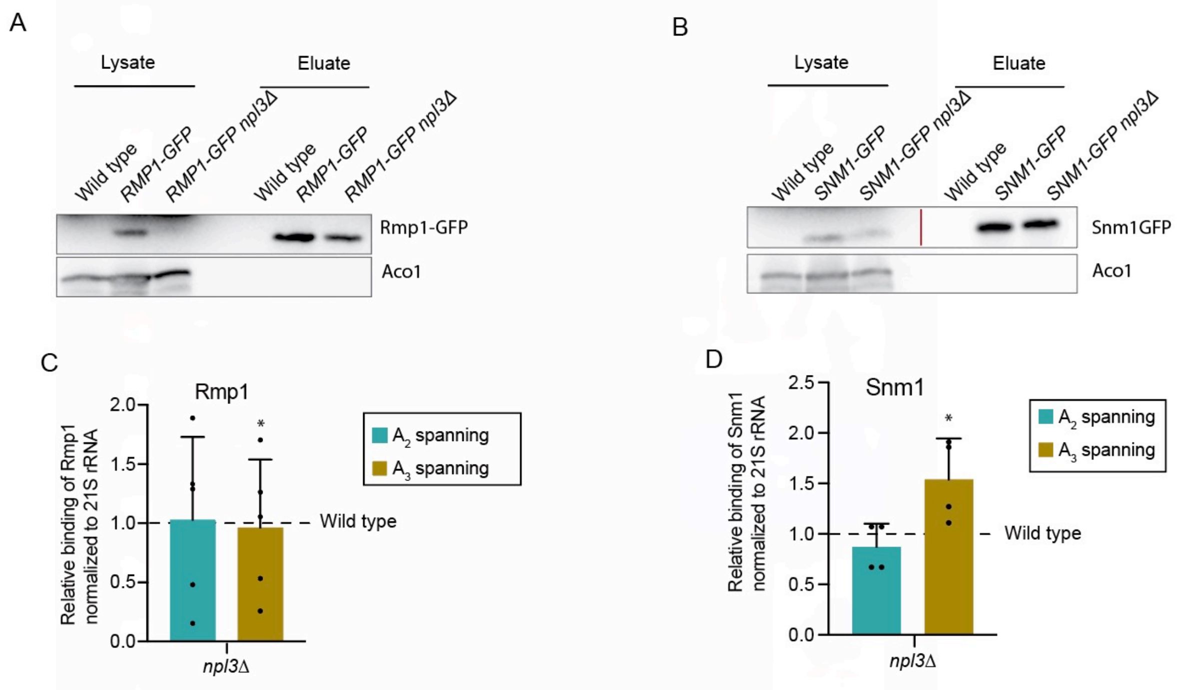

Supplementary Figure 3: (Related to Figure 5) (A, B) The western blots show an equal pull down of the precipitated proteins. RIP with GFP-tagged MRP complex components was carried out. Western blot analysis shows the pull down of GFP-tagged Rmp1 (A) and Snm1 (B). As a negative control Aco1 was used. (C, D) Subsequent qPCRs with primers amplifying a product spanning the A_2 or A_3 cleavage site followed. Binding of Rmp1 (C, n=3) and Snm1 (D, n=4) to rRNA in *npl3Δ* was related to the binding in wild type cells and normalized to mitochondrial 21S rRNA. *P < 0.05; **P < 0.01; ***P < 0.001.

Supplementary Figure 4

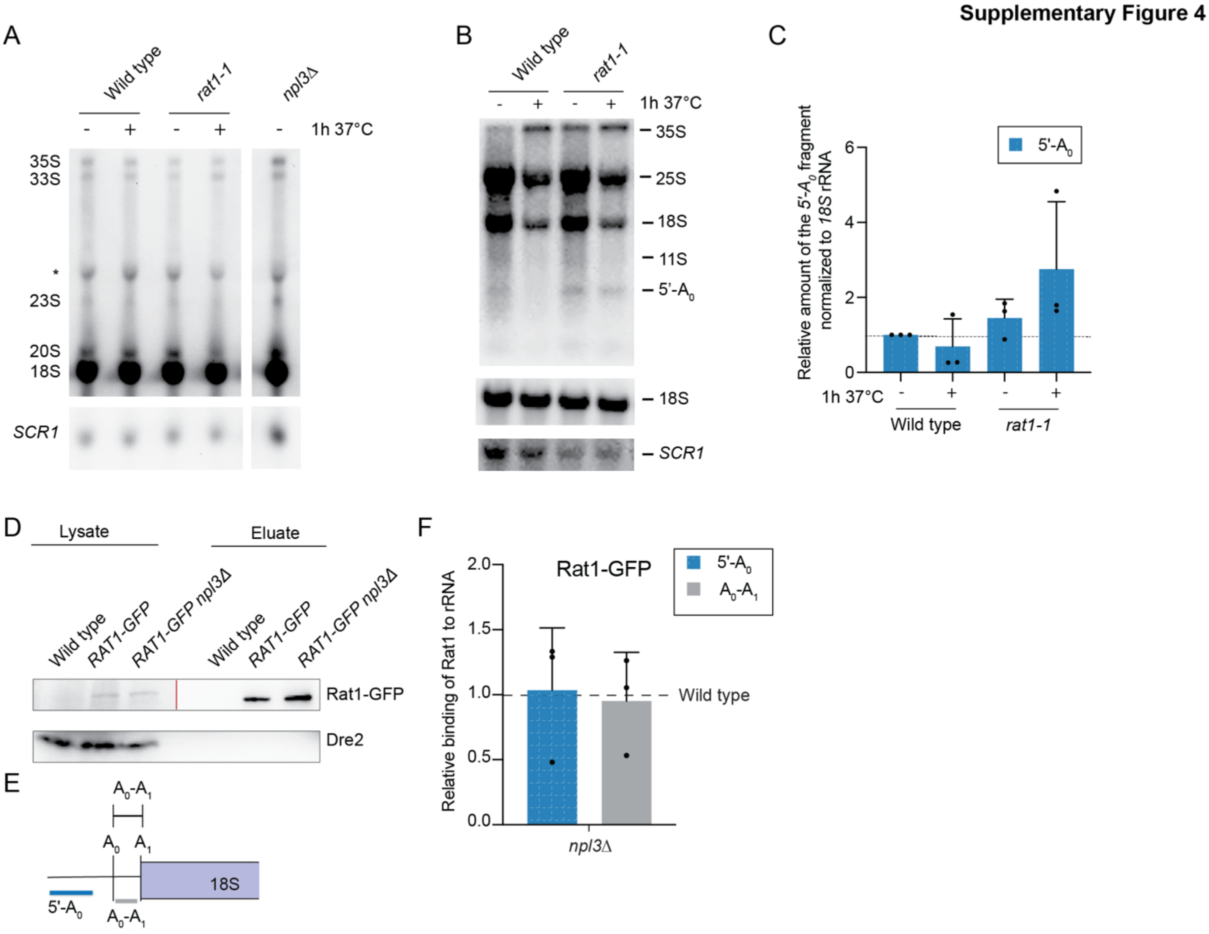

Supplementary Figure 4: (Related to Figure 6) (A) The 23S rRNA does not accumulate in *rat1-1* mutants. Northern blot analysis using the 23S rRNA-DIG probe in the temperature sensitive mutant *rat1-1* and *rat1-1 npl3Δ* was carried out after shift to 37°C for 1 h. *SCR1* served as a loading control. n=3. (B) Northern blot detecting the 5'-A_0 region of ETS1 with a digoxygenin labelled probe shows a slight accumulation of this fragment in *rat1-1* mutant cells. The *SCR1* mRNA was used as a loading control. (C) Quantification of three experiments, one of which is shown in B. (D-F) Rat1 binding to ETS1 is not altered in *npl3*Δ. RIP experiment with GFP-tagged Rat1 was carried out and analyzed on western blots (D). Dre2 served as a negative control. Subsequent qPCRs with primers binding in the ETS1 or between the A_0 and A_1 cleavage site followed. (E) The resulting fragments are indicated in blue and gray. (F) Binding to rRNA in *npl3*Δ was related to the binding in wild type cells. n=4. *P < 0.05; **P < 0.01; ***P < 0.001.

Supplementary Figure 5

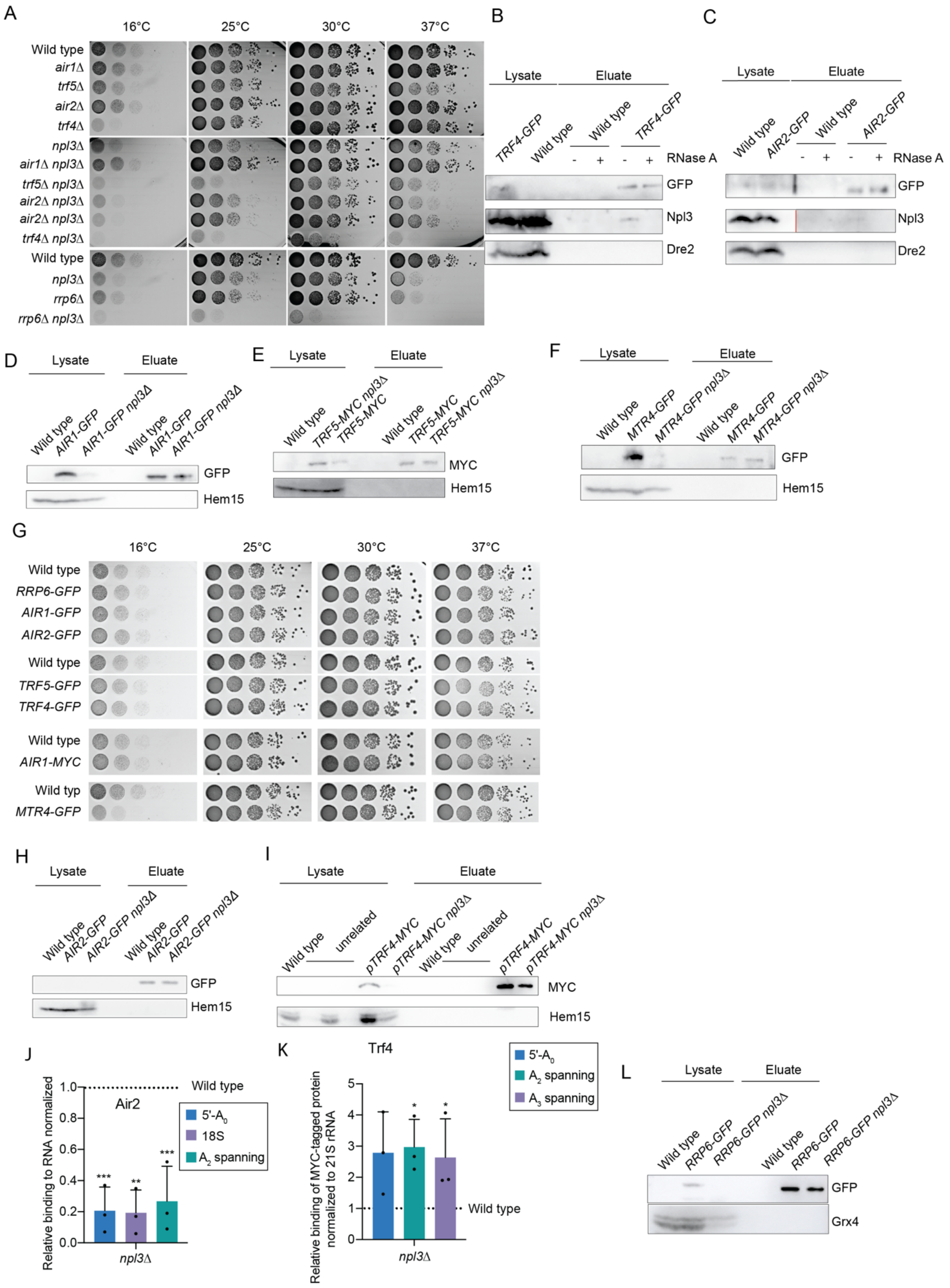

Supplementary Figure 5: (Related to Figure 6) (A) *NPL3* shows a genetic interaction with genes encoding the TRAMP complex or the nuclear exosome. Complete growth test at 16 °C, 25 °C, 30 °C and 37 °C. Part of this was shown in Figure 5A. (B, C) Npl3 physically interacts with the TRAMP-complex. Western blots of Npl3 co-IPs with and without RNase A treatment are shown for Trf4 (B) and Air2 (C). Dre2 and Hem15 served as negative controls.

n=3. (D-F) The western blots show an equal pull down of the precipitated proteins. RIP or CLIP with GFP-tagged TRAMP and exosome components was carried out. Western blot analysis shows the pull down of GFP-tagged Air1 (D, RIP), Myc-tagged Trf5 (E, RIP) and GFP-tagged Mtr4 (F, CLIP). As a negative control Hem15, Grx4, Aco1 or Dre2 were used. (A) GFP-tagged and Myc-tagged strains are functional. Growth analysis of the tagged strains is shown in comparison to wild type. The indicated endogenously tagged strains were spotted in 10-fold serial dilution onto full medium agar plates and incubated for 3 days at the indicated temperatures. (H, I) The western blots show an equal pull down of the precipitated proteins. RIP with GFP-tagged TRAMP components was carried out. Western blot analysis shows the pull down of GFP-tagged Air2 (H) and Myc-tagged Trf4 (I). (J) Air2 binding to pre-rRNA is significantly reduced when *NPL3* is deleted. RIP experiments with Air2 in wild type and *npl3*Δ and subsequent qPCRs with the indicated parts of the 35S rRNA were carried out. Relative binding to the RNA was normalized to the mitochondrial 21S rRNA. n=3. *P < 0.05; **P < 0.01; ***P < 0.001. (K) Trf4 binding to pre-rRNA is slightly elevated when Npl3 is missing. RIP experiments with Myc-tagged Trf4 were carried out (n=4). Subsequent qPCRs with indicated parts of the 35S rRNA followed. Binding was normalized to mitochondrial 21S rRNA. *P < 0.05; **P < 0.01; ***P < 0.001. (L) The western blot analysis of the CLIP with GFP-tagged Rrp6 shows an equal pull down of the precipitated proteins.

Source data 1 to Figure 1

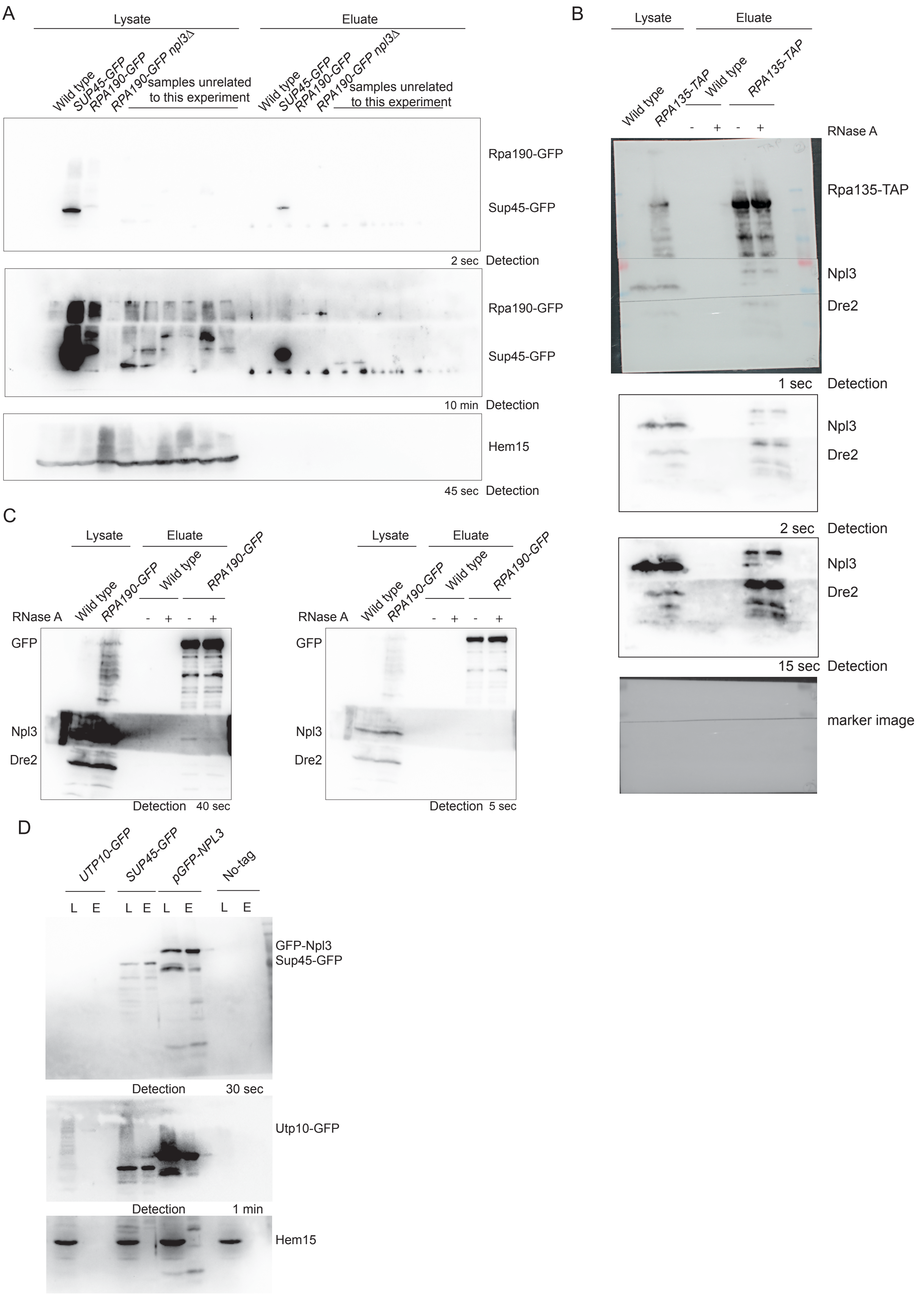

Source data 1 (Related to Figure 1): Different detection times are needed to visualize the bands on western blots. (A-D) Uncropped image with different exposure time from Figure 1C (A), E (B), F (C) and I (D). The detection time is indicated. Blots were cut horizontally to enable detection with different antibodies at the same time.

A

	Chromosome	Start	End
A0-1	chrXII	457819	457827
A1-1	chrXII	457728	457736
A2-1	chrXII	455719	455727
A3-1	chrXII	455645	455653
A0-2	chrXII	466956	466964
A1-2	chrXII	466865	466873
A2-2	chrXII	464856	464864
A3-2	chrXII	464782	464790

B

Wild type	bin 1	bin 2	bin 3	bin 4	bin 5	bin 6	bin 7	bin 8	Average
A0-1	49,81	49,81	49,79	49,77	49,57	50,1	50,01	49,5	49,795
A1-1	17,76	17,76	17,56	17,12	16,77	16,74	16,21	16,09	17,00125
A2-1	63,73	63,73	67,64	68,65	72,03	72,07	71,37	70,62	68,73
A3-1	29,48	36,86	47,81	56,72	78,44	78,37	78,92	82,74	61,1675
A0-2	29,84	29,84	29,8	29,78	29,37	30,38	30,18	29,37	29,82
A1-2	17,6	17,6	17,19	16,36	15,83	15,79	14,96	14,74	16,25875
A2-2	65,01	65,01	67,54	67,96	71,56	71,75	71,89	73,03	69,21875
A3-2	35,7	40,16	45,56	49,98	61,11	60,55	60,77	63,84	52,20875
*npl3*Δ	bin 1	bin 2	bin 3	bin 4	bin 5	bin 6	bin 7	bin 8	Average
A0-1	71,1	71,1	71,15	71,66	71,51	72,89	72,82	72,06	71,78625
A1-1	43,46	43,46	43,26	42,19	41,58	41,58	40,87	40,87	42,15875
A2-1	111	111	117,2	118,7	122,6	120,5	116,4	113,2	116,325
A3-1	46,98	58,34	79,15	98,47	144,7	144,7	145,2	152,3	108,73
A0-2	45,52	45,52	45,62	46,68	46,37	49,13	48,97	47,72	46,94125
A1-2	33,9	33,9	33,5	31,55	31	30,99	29,93	29,95	31,84
A2-2	101,4	101,4	104,8	105,3	109	107,9	106,4	106,5	105,3375
A3-2	57,33	63,62	73,74	83,34	106,9	106	105,5	110,4	88,35375

Source data 2 (Related to Figure 3): (A) Localization of cleavage sites. (B) Readcount over the cleavage sites.

A

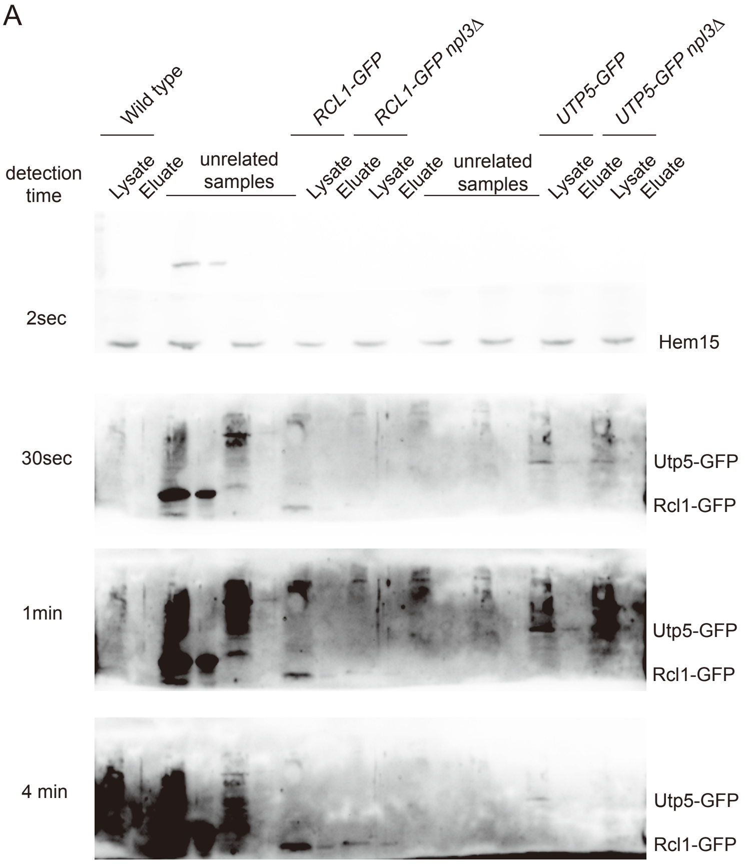

Source data 3 (Related to Figure 5): (A) Whole membrane of Figure 5P with different exposure times is shown.

Source data 4 to Figure 6

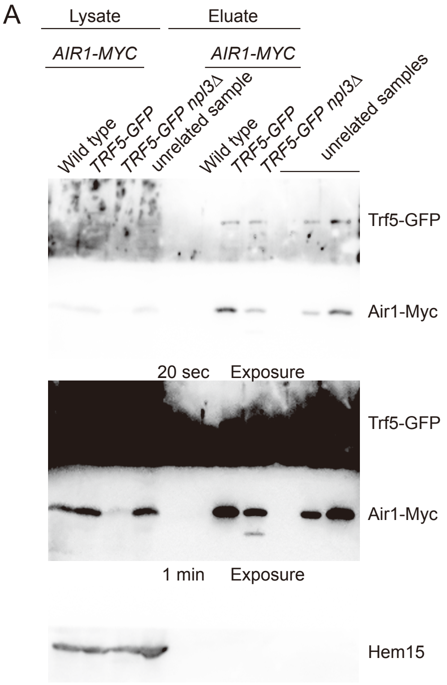

Source data 4 (Related to Figure 6): (A) Whole membrane of Figure 6J with different exposure times is shown.

Supplementary Table 1: Yeast strains

Number	Genotype	Additional genotypic information	Source
HKY36	Wild type	S288C wild type, *MATα, ura3-52 leu2Δ1 his3Δ200*	(1)
HKY189	*npl3-1*	*MATα npl3-1:URA3 leu2-1 trp1-1 his3-11*	(2)
HKY314	Wild type	*MATa his3Δ1 leu2Δ0 met15Δ0 ura3Δ0*	Euroscarf
HKY319	*rpb1-1*	*MATa rpb1-1::kanMX4* his3Δ1 leu2Δ0 ura3Δ0	This study
HKY380	*npl3Δ*	*MATa npl3::kanMX4 his3Δ1 leu2Δ0 met15Δ0 ura3Δ0*	This study
HK500	*RCL1-GFP*	*MATa RCL1-GFP:HISMX6 his3Δ1 leu2Δ0 met15Δ0 ura3Δ0*	Euroscarf
HKY682	*npl3Δ*	*MATα npl3::kanMX4 his3Δ1 leu2Δ0 ura3Δ0*	(3)
HKY694	*rpa14Δ*	*MATa rpa14::kanMX4 his3Δ1 leu2Δ0 met15Δ0 ura3Δ0*	Euroscarf
HKY1027	*rpa34Δ*	*MATα rpa34::kanMX4 his3Δ1 leu2Δ0 lys2Δ0 ura3Δ0*	Euroscarf
HKY1028	*rrp6Δ*	*MATα rrp6::kanMX4 his3Δ1 leu2Δ0 lys2Δ0 ura3Δ0*	Euroscarf
HKY1066	*RPA135-TAP*	*MATa RPA135-TAP:HISMX6 his3Δ1 leu2Δ0 met15Δ0 ura3Δ0*	Open Biosystems
HKY1112	*trf4Δ*	*MATa trf4::kanMX4 ura3Δ0 leuΔ0 his3Δ1 met15Δ0*	Euroscarf
HKY1136	*RRP6-GFP*	*MATa RRP6-GFP:HIS3MX6 his3Δ1 leu2Δ0 met15Δ0 ura3Δ0*	Invitrogen
HKY1171	*TRF4-GFP*	*MATa TRF4-GFP:HISMX6 his3Δ1 leu2Δ0 met15Δ0 ura3Δ0*	Invitrogen
HKY1196	*RRP6-GFP npl3Δ*	*RRP6-GFP:HSMX6 npl3::kanMX4 his3Δ1 leu2Δ0 met15Δ0 +pHK26 (pNPL3)*	This study
HKY1224	*rrn3-8*	*MATα rrn3-8 ade5 his7-2 leu2-112 trp1-289 ura3-52*	This study
HKY1236	*trf5Δ*	*MATa trf5::kanMX4 ura3Δ0 leuΔ0 his3Δ1 met15Δ0*	Euroscarf
HKY1237	*air1Δ*	*MATa air1::kanMX4 ura3Δ0 leuΔ0 his3Δ1 met15Δ0*	Euroscarf
HKY1238	*air2Δ*	*MATa air2::kanMX4 ura3Δ0 leuΔ0 his3Δ1 met15Δ0*	Euroscarf
HKY1304	*TRF5-GFP*	*MATa TRF5-GFP:HIS3MX6 his3Δ1 leu2Δ0 met15Δ0 ura3Δ0*	Invitrogen
HKY1309	*rrp6Δ npl3Δ*	*MATα rrp6::kanMX4 npl3::kanMX4 his3Δ1 leu2Δ0 lys2Δ0 +pHK26 (pNPL3)*	This study
HKY1373	*Rat1-GFP*	*MATa RAT1-GFP:HISMX6 his3Δ1 leu2Δ0 met15Δ0 ura3Δ0*	Invitrogen
HKY1460	*SUP45-GFP*	*MATa SUP45-GFP:HIS3MX6 his3Δ1 leu2Δ0 met15Δ0 ura3Δ0*	Invitrogen
HKY1489	*RPA190-GFP*	*MATa RPA190-GFP:HIS3MX6 leu2Δ0 met15Δ0 ura3Δ0*	Invitrogen
HKY1507	*rat1-1*	*MATa rat1-1::kanMX4 ura3Δ0 leu2Δ0 his3Δ1 met15Δ0*	This study
HKY1586	*AIR1-GFP*	*MATa AIR1-GFP:HIS3MX6 his3Δ1 leu2Δ0 met15Δ0 ura3Δ0*	Invitrogen
HKY1587	*AIR2-GFP*	*MATa AIR2-GFP:HIS3MX6 his3Δ1 leu2Δ0 met15Δ0 ura3Δ0*	Invitrogen

Number	Genotype	Additional genotypic information	Source
HKY1659	*air2Δ npl3Δ*	*MATα air2::kanMX4 npl3::kanMX4 leuΔ0 his3Δ1 met15Δ0 +pHK765 (pGFP-NPL3)*	This study
HKY1661	*trf4Δ npl3Δ*	*MATa trf4::kanMX4 npl3::kanMX4 leuΔ0 his3Δ1 met15Δ0 +pHK765 (pGFP-NPL3)*	This study
HKY1668	*AIR2-GFP npl3Δ*	*MATα AIR2-GFP:HISMX6 npl3::kanMX4 his3Δ1 leu2Δ0 met15Δ0 ura3Δ0*	This study
HKY1672	*trf5Δ npl3Δ*	*MATa trf5::kanMX4 npl3::kanMX4 leuΔ0 his3Δ1 met15Δ0 +pHK765 (GFP-NPL3)*	This study
HKY1673	*UTP18-GFP*	*MATa his3Δ1 leu2Δ0 UTP18-GFP:HIS3MX6 met15Δ0 ura3Δ0*	Invitrogen
HKY1675	*air1Δ npl3Δ*	*air1::kanMX4 npl3::kanMX4 leuΔ0 his3Δ1 +pHK765 (pGFP-NPL3)*	This study
HKY1683	*nop1-3*	*MATa nop1-3::kanMX4 ura3Δ0 leu2Δ0 his3Δ1 met15Δ0*	This study
HKY1688	*air1Δ air2Δ*	*MATα* air*1::kanMX4 air2::kanMX4 leu2-Δ1*	This study
HKY1721	*imp4-2*	*MATa imp4-2:kanMX4 ura3Δ0 leu2Δ0 his3Δ1 met15Δ0*	(4)
HKY1727	*tet::utp18*	*MATa UTP18::kanR-tet07-TATA URA3::CMV-tTA his3-1 leu2-0 met15-0*	Euroscarf
HKY1764	*RCL1-GFP npl3Δ*	*MATα RCL1-GFP::His3MX6 npl3::kanMX4 his3Δ1 leu2Δ0 met15Δ0 ura3Δ0*	This study
HKY1766	*imp4-2 npl3Δ*	*MATa imp4-2:kanMX4 npl3::kanMX4 his3Δ1 leu2Δ0 ura3Δ0*	This study
HKY1767	*nop1-3 npl3Δ*	*MATa nop1-3:kanMX4 npl3::kanMX4 ura3Δ0 leu2Δ0 his3Δ1 met15Δ0*	This study
HKY1802	*UTP5-GFP*	*MATa UTP5-GFP:HIS3MX6 his3Δ1 leu2Δ0 met15Δ0 ura3Δ0*	Invitrogen
HKY2068	*rat1-1 npl3Δ*	*rat1-1:kanMX4 npl3::kanMX4 ura3Δ0 leu2Δ0 his3Δ1*	This study
HKY2096	*air1Δ air2Δ npl3Δ*	*MATα* air*1::kanMX4 air2::kanMX4 npl3::kanMX4 leu2-Δ1 +pHK765 (GFP-NPL3)*	This study
HKY2098	*tet::utp18 npl3Δ*	*MATα UTP18::kanR-tet07-TATA URA3::CMV-tTA npl3::kanMX4 leu2-Δ1*	This study
HKY2100	*UTP5-GFP npl3Δ*	*MATα UTP5-GFP:HIS3MX6 npl3::kanMX4 leu2-Δ1*	This study
HKY2144	*utp24Δ/UTP24*	*MATa/α leuΔ0 his3Δ1 utp24::kanMX4/UTP24*	Euroscarf
HKY2146	*RRP9-GFP*	*MATa RRP9-GFP:HIS3MX6 leu2Δ0 met15Δ0 ura3Δ0*	Invitrogen
HKY2202	*RRP9-GFP npl3Δ*	*MATa RRP9-GFP:HIS3MX6 npl3::kanMX4 his3Δ1 leu2Δ0 met15Δ0 +pURA3-Npl3*	This study
HKY2253	*UTP10-GFP*	*MATa UTP10-GFP:HIS3MX6 his3Δ1 leu2Δ0 met15Δ0 ura3Δ0*	Invitrogen

Number	Genotype	Additional genotypic information	Source
HKY2268	*AIR1-GFP npl3Δ*	*MATα AIR1-GFP:HIS3MX6*<br>*npl3::kanMX4*<br>*leu2Δ0* +pHK779 (pNPL3)	This study
HKY2270	*Rat1-GFP npl3Δ*	*MATa RAT1-GFP:HISMX6*<br>*npl3::kanMX4 leu2Δ0 ura3Δ0*<br>*+pHK779*<br>(MYC-Npl3:URA3)	(3)
HKY2317	*RMP1-GFP*	*MATa RMP1-GFP:URA3*<br>*his3Δ1 leu2Δ0 met15Δ0*	This study
HKY2320	*SNM1-GFP*	*MATa SNM1-GFP:URA3*<br>*his3Δ1 leu2Δ0 met15Δ0*	This study
HKY2338	*RPA190-MYC*	*MATα*<br>*RPA190-MYC::kanMx4*<br>*his3Δ1 leu2Δ0 MET15 lys2Δ0 ura3Δ0*	(5)
HKY2484	*RPA190-MYC*<br>*UTP5-GFP*	*MATa UTP5-GFP:HIS3MX6*<br>*RPA190-MYC::kanMx4*<br>*leu2Δ0 met15Δ0 ura3Δ0*	This study
HKY2485	*RPA190-MYC*<br>*UTP5-GFP*<br>*npl3Δ*	*MATα UTP5-GFP:HIS3MX6 RPA190-*<br>*MYC::kanMx4 npl3::kanMX4 leu2Δ0*<br>*met15Δ0 ura3Δ0*	This study
HKY2517	*SNM1-GFP*<br>*npl3Δ*	*MATα SNM1-GFP::URA3*<br>*npl3::kanMX4*<br>*his3Δ1 leu2Δ0 met15Δ0*	This study
HKY2532	*utp24Δ*<br>*pMYC-UTP24*	*MATa utp24::kanMX4 leuΔ0 his3Δ1*<br>*+pHK1841(pMYC-Utp24)*	This study
HKY2539	*RMP1-GFP*<br>*npl3Δ*	*MATα RMP1-GFP:URA3*<br>*npl3::kanMX4*<br>*his3Δ1 leu2Δ0 met15Δ0*	This study
HKY2567	*MYC-SAS10*	*MATa 3xMYC-SAS10:URA3*<br>*his3Δ1 leu2Δ0 met15Δ0*	This study
HKY2568	*MYC-SAS10*<br>*npl3Δ*	*MATα 3xMYC-SAS10:URA3*<br>*npl3::kanMx his3Δ1 leu2Δ0 met15Δ0*	This study
HKY2572	*RRP9-GFP*<br>*MYC-SAS10*	*MATa 3xMYC-SAS10:URA3*<br>*RRP9-GFP:HIS3MX6 leu2Δ0 met15Δ0*	This study
HKY2573	*RRP9-GFP*<br>*SAS10-MYC*<br>*npl3Δ*	*MATa 3xMYC-SAS10:URA3*<br>*RRP9-GFP:HISMX6*<br>*npl3::kanMx4*<br>*leu2Δ0 met15Δ0*	This study
HKY2619	*AIR1-MYC*	*MATa*<br>*AIR1-MYC:URA3* his3Δ1 *leu2Δ0*<br>met15Δ0	This study
HKY2624	*AIR1-MYC*<br>*TRF5-GFP*	*MATa TRF5-GFP:HIS3MX6*<br>*AIR1-MYC:URA3 leu2Δ0*	This study
HKY2649	*AIR1-MYC*<br>*TRF5-GFP*<br>*npl3Δ*	*MATa TRF5-GFP:HIS3MX6*<br>*AIR1-MYC:URA3 npl3::KanMX4*<br>*leu2Δ0*	This study
HKY2657	*rpa190-1*	*MATa rpa190-1:kanMx4*	This study
HKY2680	*RPA190-GFP*<br>*npl3Δ*	*MATα RPA190-GFP:HIS3MX6*<br>*npl3::kanMX4 leu2Δ0 met15Δ0 ura3Δ0*	This study
HKY2833	*MTR4-GFP*	*MATa*<br>*MTR4-GFP:URA3* his3Δ1 *leu2Δ0*<br>met15Δ0	This study
HKY2834	*MTR4-GFP*<br>*npl3Δ*	*MATα MTR4-GFP:URA3*<br>*npl3::KanMX4* his3Δ1 *leu2Δ0* met15Δ0	This study
HKY2826	*UTP22-GFP*	*MATa UTP22-GFP:HIS3MX6*<br>*leu2Δ0 met15Δ0 ura3Δ0*	Euroscarf
HKY2841	*UTP22-GFP*<br>*npl3Δ*	*MATα UTP22-GFP:HIS3MX6*<br>*npl3::KanMX4 leu2Δ0 met15Δ0 ura3Δ0*	This study

Supplemental Table 2: Plasmids

Number	Genotype	Source
pHK26	*NPL3 URA3 CEN*	(2)
pHK87	*CEN LEU2 AmpR*	(6)
pHK88	*CEN URA3 AmpR*	(6)
pHK418	*GFP-NPL3 CEN LEU2*	(7)
pHK697	*RPS2-GFP URA3 CEN*	(8)
pHK765	*GFP-NPL3 CEN URA3*	(2)
pHK779	*9xMYC-NPL3 CEN ARS URA3 AmpR*	(2)
pHK1581	*GFP-NOP1 URA3 CEN AmpR*	(9)
pHK1795	*CEN yosfGFP loxP-URA3-loxP*	This study
pHK1841	*6xMYC-UTP24 URA3 AmpR CEN*	This study
pHK1903	*CEN 3xMYC loxP-URA3-loxP*	This study
pHK1924	5'UTR of *SAS10 3xMYC-SAS10 loxP-URA3-loxP*	This study

Supplemental Table 3: Primers for cloning

Construct	Number	Sequence	Name
pHK1841	HK3854	5'-GGGTGAATTTTGAGATAATTGTTGG-3'	*UTP24* 5'UTR forward
	HK3855	5'-TGCTTCCTCATTATCAGAGTTGC-3'	*UTP24* 3'UTR reverse
	HK3856	5'-TTGGAGCTCCACCGCGGTGGCGGCCGCTCTAGAACTAGTGGGGTGAATTTTGAGATAATTGTTGG-3'	*UTP24* cloning forward
	HK3856	5'-CCTCACTAAAGGGAACAAAAGCTGGGTACCGGGCCCCCCCTGCTTCCTCATTATCAGAGTTGC-3'	*UTP24* cloning reverse
	HK4597	5'-GTAACGGAAATATCGATATACTACAACAGTTAATTCCATAAGGCCTATGGGTGAAC-3'	*MYC-UTP24* RFC forward
	HK4598	5'-GCCAAACTTTCTTGTTTTCTTAGCTTTACCCATAGGCCTTCCGTTCAAGTC-3'	*MYC-UTP24* RFC reverse
pHK1924	HK5030	5'-CAATTTCACACAGGAAACAGCTATGACCATGTCTAGCAAATTATAAATACCTCAA-3'	*SAS10* forward
	HK5031	5'-ACTTTTGTTCACCCATAGAGCCTGGATTCTTGAATTTAACAGACCTCGTC-3'	*SAS10* reverse
	HK5070	5'-AGAGAAAACATATTAGAGTGGGTGTATAGAATAATATGGGTGAACAAAAGTTGATTTCTG-3'	*MYC-SAS10* forward
	HK5071	5'-TCTGTTTGAGCCTTTGCGTACCATCAAGTCTTCTTCTGAGATTAATTTTTG-3'	*MYC-SAS10* reverse

Supplemental Table 4: Primers for endogenous tagging

Strains	Number	Sequence	Template
HKY2619 HKY2624 HKY2649	HK5195	5'-CCGATCATCTTCTAACAAAAGCCAAAGAAATGGCCGTTATCTTgAACAAAAGTTGATTTCTG-3'	*AIR1-MYC* endogen fwd
	HK5196	5'-GAGAATGGAAAAAAAATTAAAAAAACTCACATATAATCCACGACGTTGTAAAACGACGGC-3'	*AIR1-MYC* endogen rev

Strains	Number	Sequence	Template
HKY2567/ HKY2568/ HKY2572/ HKY2573	HK5033	5'-GACACCACTACTTGGGTAAATACTACA TATGTAAATTTTTGTTGTAAAACGACGGC CAGTG-3'	*MYC-SAS10* endogen forward
	HK5070	5'-AGAGAAAACATATTAGAGTGGGTGTAT AGAATAATATGGGTGAACAAAAGTTGATT TCTG-3'	*MYC-SAS10* endogen reverse
HKY2317/ HKY2539	HK4528	5'-GAAAAAGAAGAACAAATCAGCCATTG ATGGCATATTCGGAatgtctaaaggcgaggaattgt ttacag-3'	*RMP1-GFP* endogen forward
	HK4529	5'-ATTGATATTATTATTTTAAAAAGTCTAT TTACTTGTATATCACGACGTTGTAAAACG ACGGC-3'	*RMP1-GFP* endogen reverse
HKY2320 HKY2517	HK4530	5'- GAAAAGTTCGTCACTGTCATTAGAAAGT TTTATGAAAAGTatgtctaaaggcgaggaattgtttac ag-3'	*SNM1-GFP* endogen forward
	HK4531	5'-AAACTTGATTTGTTATACTATATGTTCA CTGATGATACAGCACGACGTTGTAAAACG ACGGC-3'	*SNM1-GFP* endogen reverse
HKY2833 HKY2834	HK5603	5'-AATTCATAGAGATATCGTATCTGCTGGT TCTTTGTATTTAAACatgtctaaaggcgaggaattg tttacag-3'	*MTR4-GFP* endogen forward
	HK5604	5'-TTATATATACCAATAAAAATACATAATT GTGTGTGATTAACACGACGTTGTAAAACG ACGGC-3'	*MTR4-GFP* endogen reverse

Supplemental Table 5: Primers for qPCR

Number	Sequence	Name	Product
HK1867	5'-CAAACGGTGAGAGATTTCTGTGC-3'	ITS1 forward	ITS1
HK1868	5'-GCCCCGATTGCTCGAATG-3'	ITS1 reverse	
HK1879	5'-ATGCGAAAGCAGTTGAAGACAAG-3'	ETS1 forward	ETS1
HK1880	5'-CTAGGCAGATCTGACGATCACC-3'	ETS1 reverse	
HK3089	5'-AGTTACGCTAGGGATAACAGGG-3'	21S forward	21S
HK3090	5'-TGACGAACAGTCAAACCCTTC-3'	21S reverse	
HK1396	5'-CATGGCCGTTCTTAGTTGGTGG-3'	18S rRNA forward	18S
HK1397	5'-ATTGCCTCAAACTTCCATCGGC-3'	18S rRNA reverse	
HK3488	5'-TCAAACGGTGGAGAGAGTC-3'	5’ ETS1 forward	5’ ETS1
HK3489	5'- GTAATCCACCAAATCCTTCG-3'	5’ ETS1 reverse	
HK3492	5'- AGCTTTTACTGGGCAAGAAG-3'	A_2 spanning forward	A_2 spanning
HK3493	5'- GTTGCAAAGATATGAAAACTCC-3'	A_2 spanning reverse	
HK3494	5'- TGTGGAGTTTTCATATCTTTGC-3'	A_3 spanning forward	A_3 spanning
HK3495	5'- ATTACGTATCGCATTTCGC-3'	A_3 spanning reverse	
HK3534	5'- GGGTATCTGTTTGGTGGAAC-3'	A_0 spanning forward	A_0 spanning
HK3535	5'- CCACCTATTCCCTCTTGCTAG-3'	A_0 spanning reverse	
HK3536	5'- TAGCAAGAGGGAATAGGTGG-3'	A_1 spanning forward	A_1 spanning
HK3537	5'- ACGATAACTGATTTAATGAGCC-3'	A_1 spanning reverse	
HK4220	5'- GCAGTATTGAGACCATGAGAGTAG CA-3'	*NTS* forward	*Non transcribed sequence (NTS)*
HK4221	5'- TCCAAATGTAAAATGGCCTATCG-3'	*NTS* reverse	
HK5508	5'- ATGGATGGTGGCAGGCATAG-3'	*NTR* forward	*Non transcribed region (NTR)*
HK5509	5'- TCTTCCCGTCATTATCGCCC-3'	*NTR* reverse	
HK3536	5'- TAGCAAGAGGGAATAGGTGG-3'	A_1 spanning forward	A_0-A_1
HK5538	5'- CTTAAAAGAAGAAGCAACAAGC-3'	A_0-A_1 reverse	
HK1715	5'-CTGCCAGAACGCGAAATACG-3'	*QRI7* forward	*QRI7*
HK1716	5'-TTCCGGTTGTGACTTGAGCA-3'	*QRI7* forward	

Supplemental Table 6: Primers for DIG-labelling

Number	Sequence	Name
HK3689	5'-CCCGTCGCTAGTACCGATTGAATGGC-3'	23S rRNA forward
HK3690	5'-taatacgactcactatagggTTAAGCGCAGGCCCGGCTGG-3'	23S rRNA T7 reverse
HK2569	5'-GTGGGATGGGATACGTTGAG-3'	*SCR1* forward
HK2570	5'-taataggactcactataggCCAGACAGAGAGACGGATTC-3'	*SCR1* T7 reverse
HK3488	5'-TCAAACGGTGGAGAGAGTC-3'	ETS1 forward
HK3798	5'-taatacgactcactatagggGTAATCCACCAAATCCTTC-3'	ETS1 T7 reverse

Supplemental Table 7: Primers for analytical PCR

Number	Sequence	Name
HK205	5'-GTGCCCATTAACATCACC-3'	*GFP* reverse
HK423	5'-AGGCATAAATTCCGTCAGCC-3'	*KanMX4* reverse
HK690	5'-GGAATTCCATATGTCCGTTCAAGTCTTCT TCTG-3'	*MYC* reverse
HK4122	5'-ACCATAAGTGAAAGTAGTGACAAG-3'	*GFP* reverse
HK4248	5'-TCACACAGGAAACAGCTATGAC-3'	M13 reverse
HK2615	5'-ATATGCAAATGCTCGGCTCCTGTC-3'	*NPL3 5' UTR* forward
HK4683	5'-TTTGTATAGTTCATCCATGCCATGTGTAATCCTTTTT TTTTTTTTTTTTC-3'	cDNA 3'-PCR 1
HK4684	5'-TTTGTATAGTTCATCCATGCCATGTGTAATCCTTTTT TTTTTTTTTTTTG-3'	cDNA 3'-PCR 2
HK4685	5'-TTTGTATAGTTCATCCATGCCATGTGTAATCCTTTTT TTTTTTTTTTTTA-3'	cDNA 3'-PCR 3
HK3870	5'-AAACAAGAAAGTTTGGCCTCG-3'	*UTP24* seq 1 forward
HK3871	5'-TTTGATGAGCGTAGGCGGTC-3'	*UTP24* seq 2 forward
HK3872	5'-GAGGCCAAACTTTCTTGTTTTC-3'	*UTP24* seq 1 reverse
HK3873	5'-ACCGCCTACGCTCATCAAAG-3'	*UTP24* seq 2 reverse
HK4375	5'-GTTCATCCATGCCATGTGTAATCC-3'	3' PCR adapter reverse
HK5087	5'-GATTGCTGATGTGGACGCAC-3'	*SAS10* screen forward
HK5088	5'-GTGCGTCCACATCAGCAATC-3'	*SAS10* screen reverse

Supplemental Table 8: Primers for radioactive northern blot

Number	Sequence	Name
Probe a	5'-CTCTGCTGCCGGAAATGCTC-3'	5'-A_0
Probe b	5'-CACCTATTCCCTCTTGCTAG-3'	A_0-A_1
Probe c	5'-GCAGTCCACAAGCACGCCCGC-3'	25S
Probe d	5'-GAACCAAACGTCCTATTCTATTATTC-3'	18S
Probe e	5'-CGGTTTTAATTGTCCTA-3'	D-A_2

Supplemental Table 9: Antibodies for western blot and northern blot

Antibody (organism)	Dilution (method)	Source
Anti-Aco1 (rabbit)	1:2,000 (western blot)	U. Mühlenhoff, Marburg (Germany)
Anti-Digoxigenin-AP (sheep)	1:10,000 (northern blot)	Roche
Anti-Dre2 (rabbit)	1:10,00 (western blot)	U. Mühlenhoff, Marburg (Germany)
Anti-GFP (mouse)	1:5,000 (western blot)	Thermo Fisher Scientific
Anti-GFP (rabbit)	1:4,000 (western blot)	ChromoTek GmbH
Anti-GFP (rabbit)	1:10,000 (western blot)	Terry Pines Biolabs (TP401)
Anti-Grx4 (rabbit)	1:5,000 (western blot)	U. Mühlenhoff, Marburg (Germany)
Anti-Hem15 (rabbit)	1:5,000 (western blot)	U. Mühlenhoff, Marburg (Germany)
Anti-mouse IgG-HRP (goat)	1:10,000 (western blot)	Dianova
Anti-Mtr4 /ms2(rabbit)	1:2,000 (western blot)	U. Mühlenhoff, Marburg (Germany)
Anti-Myc (9E10) (mouse)	1:1,000 (western blot)	Santa Cruz
Anti-Nop1 (mouse)	1:4,000 (western blot)	EnCor Biotechnology
Anti-Npl3 (rabbit)	1:5,000 (western blot)	Heike Krebber, Göttingen (Germany)
Anti-Pgk1 (mouse)	1:10,000 (western blot)	Invitrogen
Anti-rabbit IgG-HRP (goat)	1:10,000 (western blot)	Dianova
Anti-Rpl3 (rabbit)	1:1,000 (western blot)	MyBioSource MBS9214187
Anti-Rps14 (rabbit)	1:1,000 (western blot)	Aviva Systems Biology ARP40322

SUPPLEMENTAL REFERENCES

1. Winston F, Dollard C, Ricupero-Hovasse SL. Construction of a set of convenient Saccharomyces cerevisiae strains that are isogenic to S288C. Yeast. 1995 Jan;11(1):53–5.
2. Hackmann A, Gross T, Baierlein C, Krebber H. The mRNA export factor Npl3 mediates the nuclear export of large ribosomal subunits. EMBO Rep [Internet]. 2011;12(10):1024–31. Available from: http://dx.doi.org/10.1038/embor.2011.155
3. Klama S, Hirsch AG, Schneider UM, Zander G, Seel A, Krebber H. A guard protein mediated quality control mechanism monitors 5'-capping of pre-mRNAs. Nucleic Acids Res. 2022;50(19):11301–14.
4. Li, Z., Vizeacoumar, F.J., Bahr, S., Li, J., Warringer, J., Vizeacoumar, F.S., Min, R., Vandersluis, B., Bellay, J., Devit, M. *et al.* (2011) Systematic exploration of essential yeast gene function with temperature-sensitive mutants. *Nat Biotechnol*, **29**, 361-367.
5. Albert B, Léger-Silvestre I, Normand C, Ostermaier MK, Pérez-Fernández, Panov KI, et al. RNA polymerase I-specific subunits promote polymerase clustering to enhance the rRNA gene transcription cycle. J Cell Biol. 2011;192(2):277–93.
6. Sikorski RS, Hieter P. A System of Shuttle Vectors and Yeast Host Strains Designed for Efficient Manipulation of DNA in Saccharomyces ceratisiae. Genetics. 1989;122:19–27.
7. Gilbert W, Siebel CW, Guthrie C. Phosphorylation by Sky1p promotes Npl3p shuttling and mRNA dissociation. RNA. 2001;7:302–13.
8. Milkereit P, Strauss D, Bassler J, Gadal O, Kühn H, Schütz S, et al. A Noc complex specifically involved in the formation and nuclear export of ribosomal 40 S subunits. J Biol Chem. 2003;278(6):4072–81.
9. Xu C, Henry PA, Setya A, Henry MF. In vivo analysis of nucleolar proteins modified by the yeast arginine methyltransferase Hmt1/Rmt1p. RNA. 2003 Jun;9(6):746–59.
